# Comprehensive Analysis of Microbiome, Metabolome, and Transcriptome Revealed the Mechanisms of Intestinal Injury in Rainbow Trout under Heat Stress

**DOI:** 10.3390/ijms24108569

**Published:** 2023-05-10

**Authors:** Changqing Zhou, Pan Gao, Jianlin Wang

**Affiliations:** 1 State Key Laboratory of Grassland Agro-Ecosystems, Key Laboratory of Grassland Livestock Industry Innovation, Ministry of Agriculture and Rural Affairs, Grassland Agriculture Engineering Center, Ministry of Education, College of Pastoral Agriculture Science and Technology, Lanzhou University, Lanzhou 730020, China; 2 College of Ecology, Lanzhou University, Lanzhou 730000, China

**Keywords:** rainbow trout, heat stress, gut microbiota, barrier function, multi-omics

## Abstract

Global warming is one of the most common environmental challenges faced by cold-water fish farming. Intestinal barrier function, gut microbiota, and gut microbial metabolites are significantly altered under heat stress, posing serious obstacles to the healthy artificial culture of rainbow trout. However, the molecular mechanisms underlying intestinal injury in rainbow trout under heat stress remain unclear. In the present study, the optimal growth temperature for rainbow trout (16 °C) was used for the control group, and the maximum temperature tolerated by rainbow trout (24 °C) was used for the heat stress group, which was subjected to heat stress for 21 days. The mechanism of intestinal injury in rainbow trout under heat stress was explored by combining animal histology, 16S rRNA gene amplicon sequencing, ultra-high performance liquid chromatography-mass spectrometry, and transcriptome sequencing. The results showed that the antioxidant capacity of rainbow trout was enhanced under heat stress, the levels of stress-related hormones were significantly increased, and the relative expression of genes related to heat stress proteins was significantly increased, indicating that the heat stress model of rainbow trout was successfully established. Secondly, the intestinal tract of rainbow trout showed inflammatory pathological characteristics under heat stress, with increased permeability, activation of the inflammatory factor signaling pathway, and increased relative expression of inflammatory factor genes, suggesting that the intestinal barrier function was impaired. Thirdly, heat stress caused an imbalance of intestinal commensal microbiota and changes in intestinal metabolites in rainbow trout, which participated in the stress response mainly by affecting lipid metabolism and amino acid metabolism. Finally, heat stress promoted intestinal injury in rainbow trout by activating the peroxisome proliferator-activated receptor-α signaling pathway. These results not only expand the understanding of fish stress physiology and regulation mechanisms, but also provide a scientific basis for healthy artificial culture and the reduction of rainbow trout production costs.

## 1. Introduction

Global warming is one of the most common environmental challenges faced by cold-water fish farming. The ability of fish to cope with environmental variation is considered a major determinant of fish health [1]. It is predicted that ocean temperatures will increase by 1–4 °C by 2100, which will have potential effects on stress physiology and stress regulation in fish [1]. Rising water temperatures are devastating for fish, especially for cold-water fish. Because fish are ectotherms, they are particularly vulnerable to changes in environmental temperature. Water temperatures that are too high or too low can cause physiological dysfunction or even death of fish [2]. Global warming is limiting aquaculture development by affecting fish survival through chronic temperature increases and an increased frequency of extreme heatwave events, while affecting fishery ecosystems and economic benefits.

Rainbow trout (*Oncorhynchus mykiss*) is a species of cold-water salmonid fish native to the Pacific coast countries of North America, with essential economic and research value [3]. Rainbow trout is one of the four major freshwater aquaculture fish recommended by the Food and Agriculture Organization of the United Nations (FAO) and is widely cultivated as an economic fish worldwide. It has the characteristics of fresh meat, no intermuscular spines, high protein, and low cholesterol, which play a vital role in improving human dietary structure. Rainbow trout have high water quality requirements, and the water should not only be clear and contain high oxygen levels, but also have low temperature, generally between 14 °C to 18 °C. Rainbow trout farmed in open-water cages, inland tanks, and ponds are vulnerable to rising water temperatures from seasonal fluctuations and climate change [4]. In order to survive, fish will adjust and change at various levels, such as behavior, physiology, tissues, cells, and molecules, along with environmental changes [5,6]. Stress responses occur when water temperatures exceed 20 °C, which can lead to reduced food intake, abnormal behavior, growth inhibition, and even death [3,5]. Domestic and foreign studies on heat stress in rainbow trout mainly focus on oxygen consumption changes, stress hormone regulation, growth inhibition, heat stress proteins (HSPs), immune factors, and innate immunity [4,5,6,7,8]. Recent studies have found that when water temperature exceeds optimal growth temperature, it can induce a stress response, resulting in intestinal damage and decreased abundance and diversity of intestinal microbiota [4,6]. An increasing number of studies have shown that increased environmental temperatures reduce the relative abundance and diversity of gut microbiota in animals, which may have more negative effects on hosts, such as Chinese giant salamander [9], red-back salamander [10], common lizards [11], and laying hens [12]. Exposure to stressors can lead to changes in the brain–gut axis (BGA) of animals (such as fish, humans, and other mammals), eventually leading to a series of inflammatory responses and intestinal diseases [13,14,15,16,17]. Related studies have shown that stress can lead to gastrointestinal dysfunction, as well as intestinal inflammation and infection [14,16,18]. Several biomarkers, including HSPs, tight junctions (TJs), cytokines, oxidative stress factors, and their tissue expression levels, are commonly used to assess intestinal injury caused by heat stress [19]. However, the mechanism of intestinal injury in fish under heat stress is still not fully understood.

The analytical approaches of single omics, including microbiome, metabolome, and transcriptome, are mature and widely used in the fields of biology, medicine, pharmacy, and agronomy. The comprehensive use of gut microbiome, transcriptome, and metabolome technologies is considered as the most effective research method to assess environment–host–microbiome interactions [20]. However, the physiological regulation of intestinal injury in rainbow trout under heat stress remains a complex scientific issue, and no single omics can systematically reveal the mechanism. The combination of multiple omics is helpful for a further understanding of the regulatory mechanism of intestinal injury in rainbow trout under heat stress from a systematic and holistic perspective. In the present research, we investigated the effects of heat stress on intestinal function in rainbow trout and further explored the mechanism of intestinal injury by combined analysis of microbiome, metabolome, and transcriptome. This study may provide a theoretical basis for analyzing the mechanism of intestinal injury in rainbow trout under heat stress.

## 2. Results

### 2.1. Detection of Stress Markers

**Figure 1:**
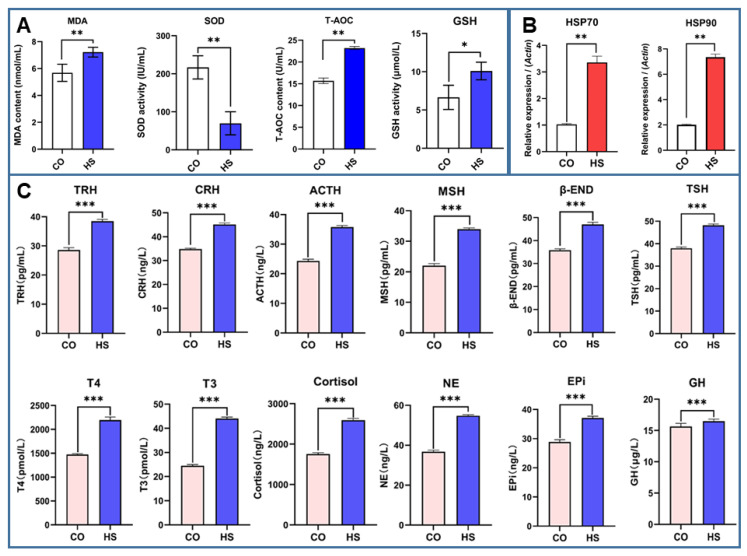
Changes in stress indices in rainbow trout. (**A**). The antioxidant indices of intestinal contents measured by ELISA included MDA, SOD, T-AOC, and GSH. (**B**). Serum levels of stress hormones were measured by ELISA, including TRH, CRH, ACTH, MSH, β-END, TSH, T4, T3, Cortisol, NE, Epi, and GH. (**C**). The expression of *HSP70* and *HSP90* mRNA in intestine was determined by qRT-PCR. The reference gene is *β-actin*. Bars represent geometric means ± SD. N = 3 biological replicates. *, *p* < 0.05; **, *p* < 0.01; ***, *p* < 0.001 using a one-way analysis of variance (ANOVA) followed by Tukey’s HSD test.

The antioxidant factors in the intestinal contents of rainbow trout were measured by ELISA under heat stress (Figure 1). The results showed that the levels of MDA, T-AOC, and GSH in intestinal contents were significantly increased, while the level of SOD was significantly decreased, indicating that there was a large accumulation of peroxides in intestinal contents under heat stress, which may cause oxidative damage.

To investigate changes in hormone levels in rainbow trout under heat stress, stress-related hormone levels in rainbow trout serum were measured by ELISA (Figure 1C). It was found that the hypothalamic–pituitary–adrenal (HPA) axis and hypothalamic–sympathetic–chromaffin cell (HSC) axis were activated and the levels of hormones related to stress regulation were significantly increased in rainbow trout. The serum levels of TRH, CRH, ACTH, MSH, β-END, TSH, T4, T3, Cortisol, NE, Epi, and GH in rainbow trout under heat stress were significantly higher than those in the CO group. These results suggest that neuroendocrine function is involved in adaptive regulation in a high temperature environment, and the enhanced activity of neuroendocrine function induced by heat stress may be related to metabolic dysfunction. HSPs are one of the most vital biomarkers of the heat stress response. The relative expression of *HSP90* and *HSP70* mRNA was significantly increased in the intestinal tissues of rainbow trout under heat stress (Figure 1B). The results further indicated that the heat stress model of rainbow trout was successfully established.

### 2.2. Effects of Heat Stress on Intestinal Barrier Function

To understand the effect of heat stress on the ultrastructure of the intestinal mucosal surface of rainbow trout, the intestinal tissues of rainbow trout were studied by SEM (Figure S1). The results showed that the morphology of the intestinal mucosal surface was blurred, the morphology of the intestinal villi was obviously damaged, the spacing between plica was significantly widened, and the diameter of secretory pores on the surface of epithelial cells was increased. These results indicate that the intestinal structure of rainbow trout is damaged under heat stress, and the normal function of the intestine may be affected.

**Figure 2:**
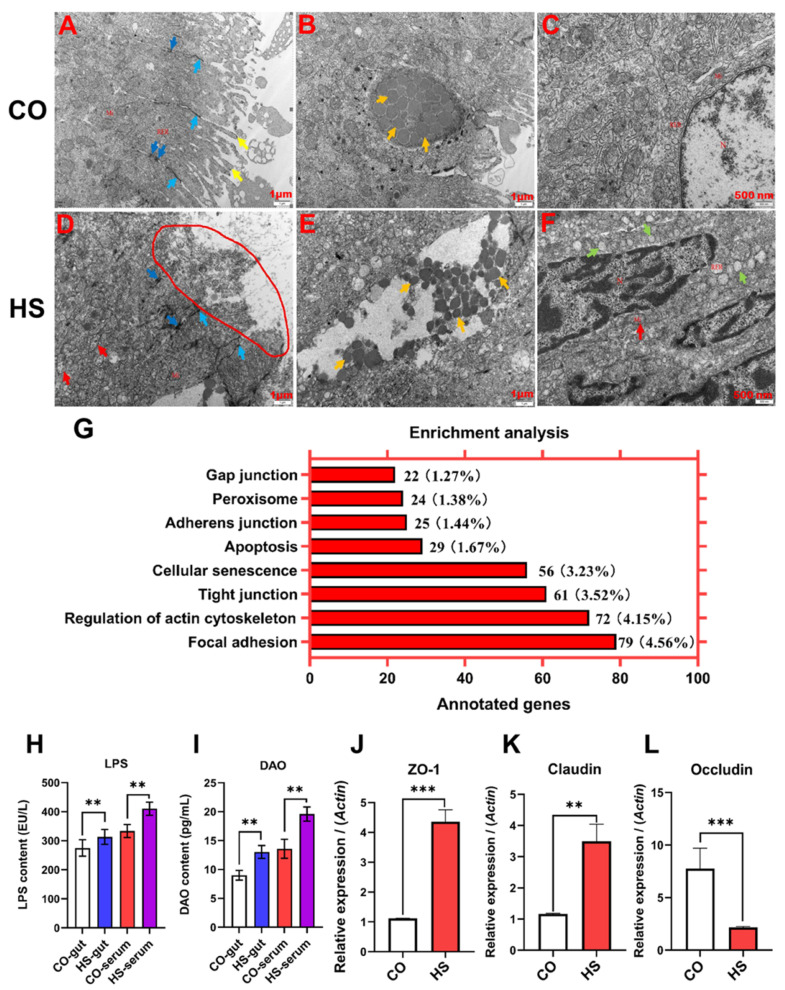
The intestinal barrier function is damaged under heat stress. (**A**–**F**). The intestinal morphology (distal gut) of the CO group and HS group was observed under TEM; microvilli (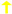); mitochondria (Mi); rough endoplasmic reticulum (RER); tight junction (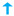); desmosome junction (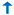); mitochondrial structure ambiguity (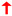); absorptive cell (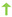); secretory granules (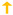); cell nucleus (N);there was no obvious microvillus structure on the free surface of cells (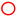);. (**G**) The altered intestinal permeability signaling pathways in the HS groups compared with CO groups by enrichment analysis. (**H**,**I**). The effect of LPS and DAO in serum and intestinal contents on intestinal permeability was determined by ELISA. (**J**–**L**). The relative mRNA expression abundance of *ZO-1*, *Occludin*, and *Claudin* in the intestine was detected by qRT-PCR. The reference gene is *β-actin*. Data are expressed as means ± SD. **, *p* < 0.01; ***, *p* < 0.001 using a one-way analysis of variance (ANOVA) followed by Tukey’s HSD test. CO, control groups; HS, heat stress groups.

The ultrastructure of intestine of rainbow trout under heat stress was studied by TEM (Figure 2A–F). The results showed that the overall microscopic cellular structure of rainbow trout gut was significantly damaged under heat stress. The nuclei of the absorbed cells were slightly deformed, the chromatin in the nucleus was aggregated, and there were large numbers of dilated rough endoplasmic reticula in the cytoplasm. There were no obvious microvilli on the cell-free surface. The tight junction and desmosome junction structures were damaged. Microvilli of intestinal epithelial cells were disrupted and shed. Endocrine granules of goblet cells were dissolved and lost. The size and number of mitochondria increased, and the cristae became shorter, fractured, dissolved, and even vacuolated. The rough endoplasmic reticulum was edematous, the ribosomal surface particles were shed, and the cyst cavity expanded and rounded into vesicles of varying sizes. Therefore, TEM further confirmed that the intestinal morphology and ultrastructure of rainbow trout were damaged under heat stress.

To investigate the effects of heat stress on intestinal barrier function in rainbow trout, LPS and DAO levels in serum and intestinal contents were measured by ELISA (Figure 2H,I). The results showed that the serum levels of LPS and DAO were significantly increased in rainbow trout under heat stress. The increased intestinal permeability suggests that heat stress may cause changes in the intestinal barrier structure of rainbow trout.

To investigate the effects of heat stress on the intestinal mucosal barrier function of rainbow trout, the intestinal mucosa of rainbow trout was studied by transcriptome sequencing technology and functional enrichment analysis (Figure 2G). The results showed that the DEGs related to intestinal permeability were significantly enriched in eight functional pathways: focal adhesion, regulation of actin cytoskeleton, tight junction, cellular senescence, apoptosis, adherens junction, peroxisome, and gap junction. The results showed that the relative expression abundance of zonula occludens 1 (*ZO-1*) and *claudin* mRNA in the intestine of rainbow trout increased significantly under heat stress, while the relative expression abundance of *occludin* mRNA decreased significantly (Figure 2J–L). These results indicate that heat stress increases intestinal permeability by regulating the expression of intestinal tight junction-related genes.

### 2.3. Heat Stress Promotes the Intestinal Inflammatory Response

**Figure 3:**
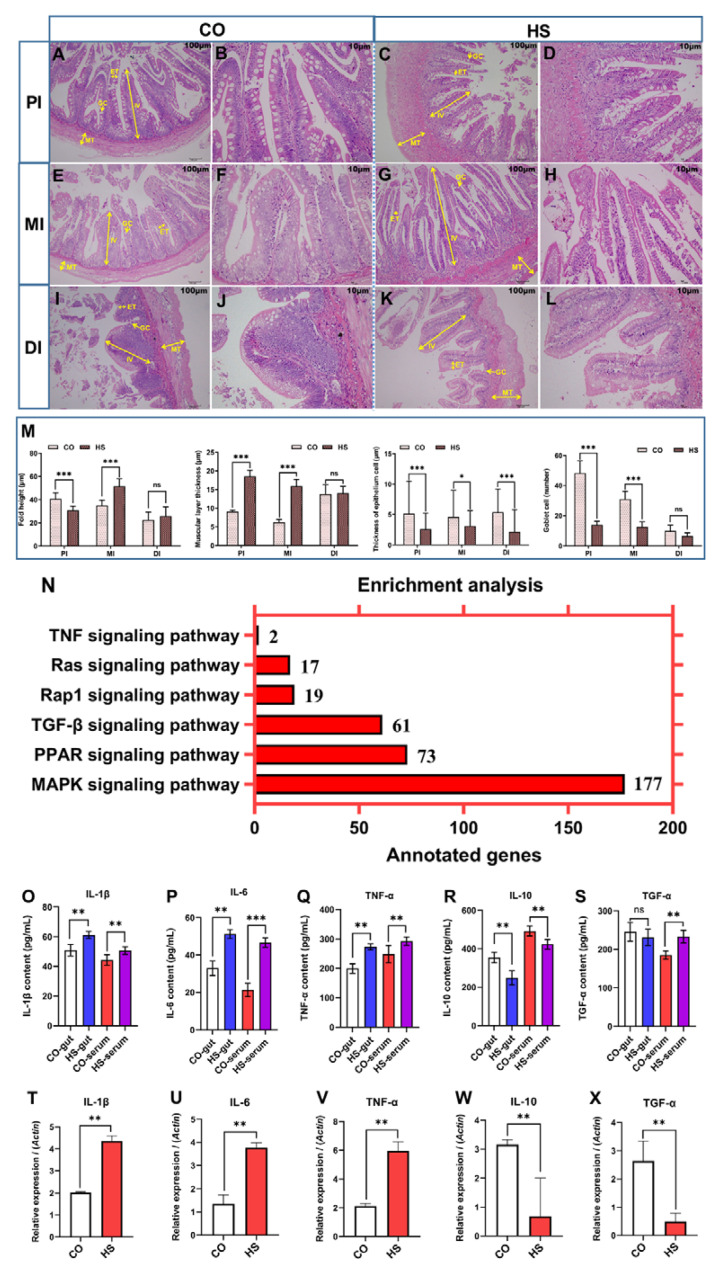
Changes in intestinal inflammatory response under heat stress. (**A**–**L**) Morphological changes in the proximal gut, mid gut, and distal gut under heat stress. IV, length of intestinal villi (N = 15); MT, muscular layer thickness (N = 10); ET, the thickness of epithelium (N = 25); GC, goblet cell number. (**M**) The measured gut IV, MT, ET, and GC, respectively. (**N**) The altered intestinal inflammatory signaling pathways, by enrichment analysis. (**O**–**S**) The levels of cytokines, including IL-1β, IL-6, TNF-α, IL-10, and TGF-α, in intestinal contents and serum were measured by ELISA. CO-gut, intestinal contents of the control group; CO-serum, serum of the control group; HS-gut, intestinal contents of the heat stress group; HS-serum, serum of the heat stress group. (**T**–**X**) The mRNA levels of *IL-1β*, *IL-6*, *TNF-α*, *IL-10*, and *TGF-α* in the intestinal were detected by qRT-PCR. The reference gene is *β-actin*. Data are expressed as means ± SD. ns, no significance; *, *p* < 0.05; **, *p* < 0.01; ***, *p* < 0.001 using a one-way analysis of variance (ANOVA) followed by Tukey’s HSD test. CO, control groups; HS, heat stress groups.

To investigate the pathological changes in the gut under heat stress, the proximal gut, mid gut, and distal gut of rainbow trout were studied by paraffin sections and H&E staining (Figure 3A–L). The results showed that the intestinal mucosal epithelium had obvious degeneration, necrosis, and exfoliation. There were red blood cell infiltration and varying degrees of inflammatory exudation in the lamina propria, submucosa, and muscle layer. The lamina propria, submucosa, and muscularis were thickened with marked inflammatory edema.

According to histological measurements and statistics, there were significant changes in the height and width of intestinal folds under heat stress (Figure 3M). The height of the proximal gut fold was significantly decreased (*p* < 0.01), and the height of the mid gut fold was significantly increased (*p* < 0.01), while the height of the distal gut fold was not significantly different. Under heat stress, the muscular layer thickness of the proximal gut and mid gut increased significantly (*p* < 0.05), while the thickness of the distal gut did not change significantly. The thickness of intestinal epithelial cells was significantly decreased under heat stress (*p* < 0.05), and the difference was extremely significant between the proximal gut and distal gut (*p* < 0.01). The number of goblet cells per unit area in the proximal and mid gut was significantly decreased under heat stress (*p* < 0.01), while the number of goblet cells in the distal gut was not significantly changed. Therefore, the intestine of rainbow trout showed obvious pathological damage under heat stress.

A semi-quantitative scoring system was used to assess the levels of inflammation in the proximal gut, mid gut, and distal gut of rainbow trout under heat stress (Table 1). The results showed that compared with the control group, the inflammation index of lamina propria, villus damage index, and tissue edema index of the proximal gut, mid gut, and distal gut were significantly increased under heat stress. These results suggested that there were obvious inflammatory responses and epithelial cell shedding characteristics in the intestine of rainbow trout under heat stress.

To characterize the expression of genes involved in inflammatory signaling pathways in the intestinal mucosa under heat stress, transcriptome sequencing was performed on the intestinal mucosa of rainbow trout. KEGG functional enrichment analysis of DEGs showed that TNF, transforming growth factor (TGF-β), mitogen-activated protein kinase (MAPK), peroxisome proliferator-activated receptor (PPAR), Rap1, and Ras signaling pathway were activated by heat stress (Figure 3N). The results showed that heat stress enhanced inflammatory responses in the gut of rainbow trout.

In order to verify the DEGs in inflammation-related pathways and the levels of inflammatory cytokines in the intestine (Figure 3O–X), the levels of pro-inflammatory cytokines IL-1β, IL-6, and TNF-α in the intestinal contents and serum were measured and found to be significantly increased. The level of anti-inflammatory cytokine IL-10 in intestinal contents and serum was significantly decreased, and the anti-inflammatory cytokine TGF-α was significantly increased in serum and slightly decreased in intestinal contents. These results indicate that the innate immune function of rainbow trout gut is activated under heat stress, and the inflammatory response exists in rainbow trout gut under heat stress, which may be related to oxidative stress injury. The results of qRT-PCR showed that compared with the CO group, the mRNA relative abundance of pro-inflammatory cytokines *IL-1β*, *IL-6*, and *TNF-α* genes was significantly increased, while the mRNA relative abundance of anti-inflammatory cytokines *TGF-α* and *IL-10* genes was significantly decreased in rainbow trout under heat stress. These results suggest that heat stress increases the expression of pro-inflammatory cytokine genes and decreases the expression of anti-inflammatory cytokine genes, thereby exacerbating the inflammatory response in the intestine of rainbow trout.

### 2.4. Heat Stress Induces Dysbiosis of Intestinal Commensal Microbiota

**Figure 4:**
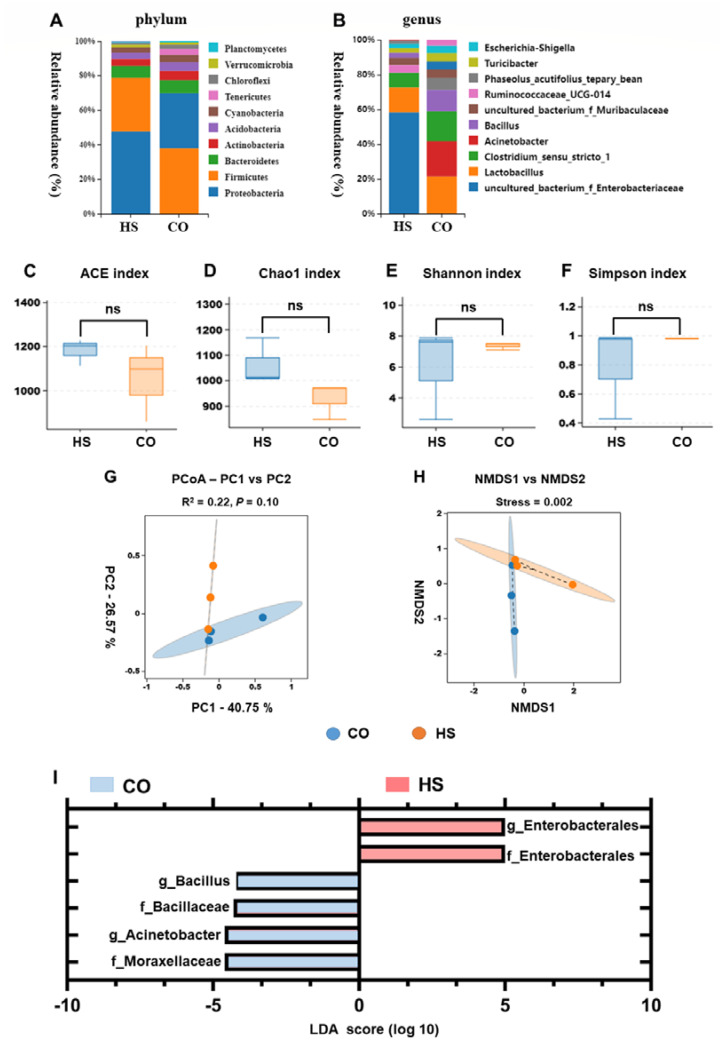
The intestinal commensal microbiota of rainbow trout is altered under heat stress. (**A**) Changes in the composition of gut microbiota at the phylum levels under heat stress. (**B**) Changes in the composition of gut microbiota at the genus levels under heat stress. (**C**–**F**) Analysis of alpha diversity of intestinal microbiota under heat stress. (**C**) ACE index; (**D**) Chao1 index; (**E**) Shannon index; (**F**) Simpson index; ns, no significance. (**G**–**I**) Analysis of beta diversity of intestinal microbiota under heat stress. (**G**) PCoA; (**H**) NMDS; (**I**) differential bacteria obtained by LEfSe with the threshold of LDA > 4 and *p* < 0.05. CO, control groups; HS, heat stress groups.

To investigate the effects of heat stress on the intestinal commensal microbiota of rainbow trout, the composition of the microbial community in the intestinal contents was analyzed by 16S rRNA amplicon gene sequencing. At the phylum level, Proteobacteria, Firmicutes, and Bacteroidetes were the dominant phyla in the gut contents of rainbow trout (Figure 4A,B). The results showed that the proportion of Proteobacteria in intestinal contents increased from 30.59% in the CO group to 46.53% in the HS group. The proportion of Firmicutes decreased from 36.24% in the CO group to 30.17% in the HS group. The proportion of Bacteroidetes decreased from 7.31% in the CO group to 6.78% in the HS group. At the genus level, *Enterobacteriaceae*, *Lactobacillus*, *Clostridium*, *Acinetobacter*, and *Bacillus* were the dominant species in the intestinal contents of rainbow trout. The results showed that the proportion of *Enterobacteriaceae* in intestinal contents increased from 1.78% in the CO group to 33.79% in the HS group. The proportion of *Lactobacillus* increased from 7.94% in the CO group to 8.38% in the HS group. The proportion of *Clostridium* decreased from 6.44% in the CO group to 4.90% in the HS group. The proportion of *Acinetobacter* decreased from 7.54% in the CO group to 0.32% in the HS group. The proportion of *Bacillus* decreased from 4.54% in the CO group to 1.77% in the HS group. The results showed that heat stress significantly affected the composition of the intestinal commensal microbiota in rainbow trout.

The alpha diversity index of the gut contents microbiota was assessed by using a *t*-test (Figure 4C–F). According to the alpha diversity index, the Chao1 index in the intestinal microflora of rainbow trout was significantly increased under heat stress (*p* < 0.05). The Chao1, ACE, and PD-whole tree indices increased slightly, but the differences were not significant. The Shannon and Simpson indices decreased slightly, but the differences were not significant. Therefore, the alpha-diversity analysis indicated that heat stress had little effect on the diversity of the microbiota in the intestinal contents of rainbow trout.

The intestinal contents of rainbow trout subjected to heat stress were analyzed for beta diversity by using the Bray-Curtis algorithm (Figure 4G,H). The PCoA showed that the first and second principal components of gut microbiota under heat stress were 40.75% and 26.57%, respectively. These results indicated that heat stress significantly altered the composition of gut microbiota in rainbow trout. The NMDS analysis showed that the stress values were less than 0.2, which further indicated that the difference between the groups had certain reliability. In addition, PERMANOVA was used to test for significant differences in beta diversity between groups. The results showed that the R^2^ values in PCoA and NMDS were all greater than 0.22, indicating that the differences between groups were large and the reliability was high.

LefSe analysis was used to screen biomarkers with significant differences between groups from phylum level to genus level (LDA score > 4) (Figure 4I). The results showed that a total of 11 strains of differential microflora were screened from the intestinal contents, including two orders, three families, three genera, and two species. *Bacillus*, Bacillaceae, *Acinetobacter*, and Moraxellaceae were the lowest abundance markers in intestinal contents under heat stress. Enterobacterales were the most abundant markers under heat stress.

### 2.5. Heat Stress Alters Gut Microbiota-Associated Metabolites

**Figure 5:**
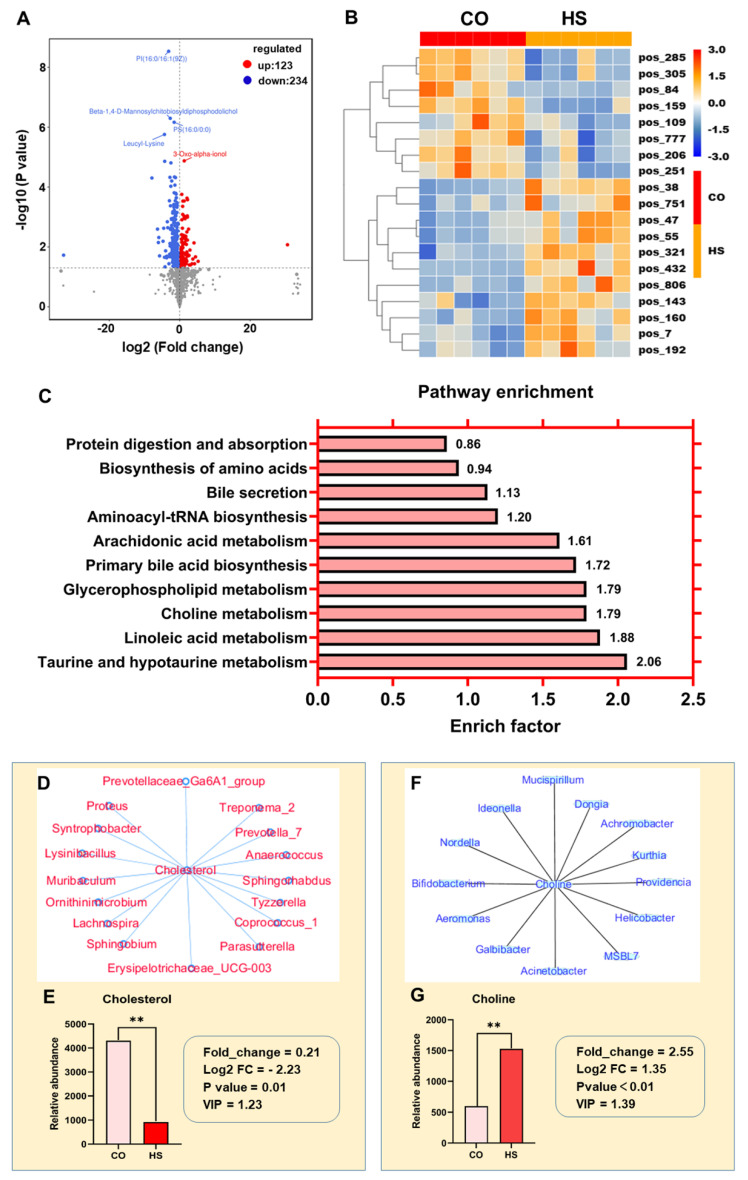
Heat stress alters gut microbiota-related metabolites in rainbow trout. (**A**) Volcano plot of differential metabolites in the gut under heat stress. (**B**) Heat map of differential metabolites in the gut under heat stress. (**C**) KEGG pathway enrichment analysis of differential metabolites in the gut under heat stress. (**D**,**E**) Spearman correlation was used to analyze the association between bacteria and differential metabolites (cholesterol): (**D**) the correlation network diagram of metabolites and gut microbiota; (**E**) the relative expression level of the differential metabolite cholesterol. (**F**,**G**) Spearman correlation was used to analyze the association between bacteria and differential metabolites (choline): (**F**) the correlation network diagram of metabolites and gut microbiota; (**G**) the relative expression level of the differential metabolite choline. Correlation coefficient (CC) > 0.8 or < −0.8. **, *p* < 0.01 using a one-way analysis of variance (ANOVA) followed by Tukey’s HSD test.

The differential metabolites in the intestinal contents of rainbow trout under heat stress were investigated by UPLC-MC and OPLS-DA. The results showed that the metabolic profile of intestinal contents changed significantly under heat stress (Figure 5A). Compared with the CO group, 357 metabolites (123 up-regulated and 234 down-regulated) were changed in the positive ion pattern. KEGG enrichment analysis showed that the differential metabolites were significantly enriched in glycerophospholipid metabolism, choline metabolism, linoleic acid metabolism, taurine and hypotaurine metabolism, bile secretion, and arachidonic acid metabolism signaling pathways (Figure 5C). The pathways of bile secretion and primary bile acid biosynthesis were most enriched in the intestine of rainbow trout under heat stress. Under heat stress, the abundance of cholesterol in intestinal contents was significantly reduced. On the one hand, the synthesis and secretion of bile acids were decreased, and the process of lipid metabolism was affected. On the other hand, it changed the fluidity of the cell membrane and affected the normal physiological function of the cell membrane.

To determine the potential association between microbiota and metabolites, correlation analysis between gut microbiota and metabolites was performed using Spearman’s correlation method (Figure 5D,E). The results showed that five gut microbiota species were positively correlated with cholesterol metabolites (correlation coefficient > 0.8). These bacteria included *Lysinibacillus*, *Proteus*, *Anaerococcus*, *Sphingorhabdus*, and *Ornithinimicrobium*. Eleven gut bacteria were negatively correlated with cholesterol metabolites (correlation coefficient > −0.8), including *Prevotella_7*, *Syntrophobacter*, *Parasutterella*, *Muribaculum*, *Treponema_2*, *Prevotellaceae_Ga6A1_group*, *Sphingobium*, *Lachnospira*, *Erysipelotrichaceae_UCG-003*, *Tyzzerella*, and *Coprococcus_1*. Therefore, the intestinal microbial imbalance and metabolite changes in rainbow trout under heat stress may jointly affect the lipid metabolism process and the normal physiological function of the cell membrane.

Glycerophospholipid metabolic pathways were the most enriched in the intestine of rainbow trout under heat stress. Under heat stress, the abundance of choline in intestinal contents was significantly reduced. Gut microbiota was correlated with metabolites by Spearman’s correlation (Figure 5F,G). The results showed that *Galbibacter* and *Ideonella* had the highest positive correlation coefficient with choline, while *Helicobacter*, *Acinetobacter*, *Achromobacter*, *Aeromonas*, *Kurthia*, *Providencia*, *Bifidobacterium*, and *Mucispirillum* were negatively correlated with choline. Therefore, gut microbial imbalance and altered metabolites may act together to cause intestinal inflammation and injury.

### 2.6. Heat Stress Induces Intestinal Injury by Activating PPAR-α Signaling Pathway

**Figure 6:**
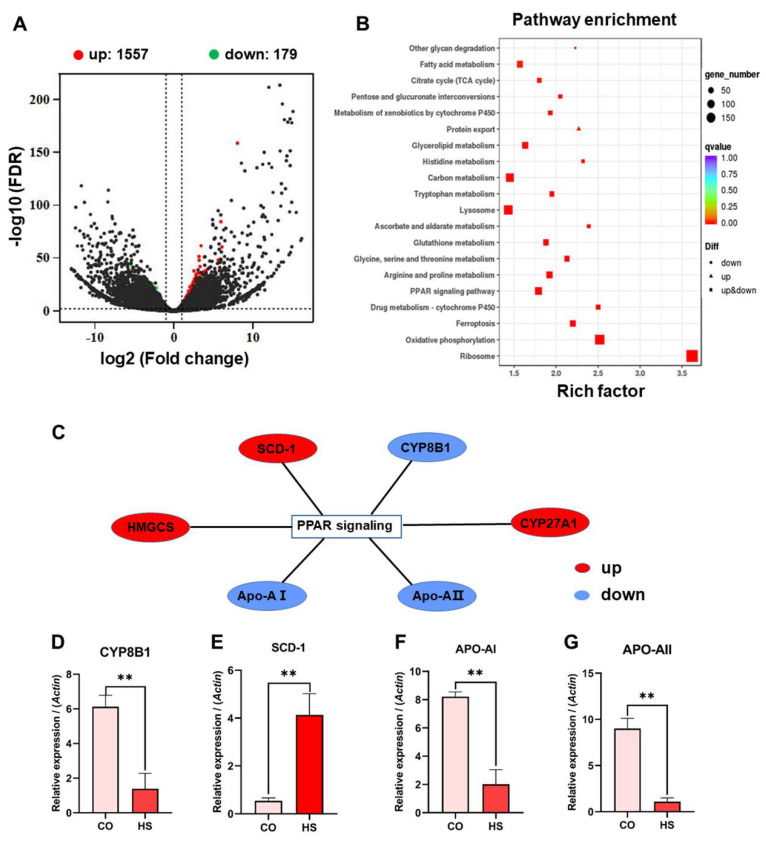
Heat stress induces intestinal injury by activating the PPAR-α signaling pathway. (**A**) Volcano plot of DEGs in intestinal mucosa under heat stress; The black dots indicate no significant difference. (**B**) KEGG pathway enrichment analysis of DEGs in intestinal mucosa under heat stress. (**C**) Enrichment analysis of PPAR-α signaling pathway. (**D**–**G**) The DEGs in intestinal mucosa were verified by qRT-PCR. The reference gene is *β*-*actin*. Data are expressed as means ± SD. **, *p* < 0.01 using a one-way analysis of variance (ANOVA) followed by Tukey’s HSD test. CO, control groups; HS, heat stress groups.

In order to explore the molecular mechanism of heat stress on the intestinal injury of rainbow trout, we used transcriptome sequencing technology to analyze the enrichment of DEGs and pathways in the intestinal mucosa of rainbow trout under heat stress. The results showed that there were 1736 DEGs (FDR < 0.01; fold change > 2 or <−2) in the intestinal mucosa of rainbow trout under heat stress, of which 1557 were up-regulated and 179 were down-regulated (Figure 6A). Enrichment analysis showed that the PPAR signaling pathway was activated in the intestinal mucosa of rainbow trout under heat stress, and 73 DEGs were enriched in this signaling pathway (Figure 6B,C). The activation of the PPAR signaling pathway is usually associated with the lipid metabolic process.

Under heat stress, PPAR-α activation down-regulated target gene cytochrome P450 8B1 (*CYP8B1*) through the adipocytokine signaling pathway, which affected cholesterol metabolism. It affected lipogenesis by up-regulating the target gene stearoyl-CoA desatu-rase-1 (*SCD-1*). It also affected the lipid transport process by down-regulating the target genes apolipoprotein A-I (*Apo-AⅠ*) and apolipoprotein A-Ⅱ (*Apo-AⅡ*) (Figure 6D–G). These results indicated that the fatty acid production of rainbow trout was enhanced, while the cholesterol metabolism and lipid transport ability were decreased under heat stress. 

## 3. Discussion

### 3.1. Heat Stress Model of Rainbow Trout Established

Most fish are ectotherms. Under heat stress, due to the production and accumulation of a large number of free radicals and reactive oxygen species (ROS), the antioxidant capacity of the body is reduced, which in turn causes oxidative stress and leads to oxidative damage of tissue and cells [21]. Some studies have suggested that there is a link between heat stress and oxidative stress in animals exposed to a heat environment and oxidants because the expression of *HSPs* and antioxidant enzymes genes is consistent. In the present study, heat stress caused a significant increase in the levels of MDA, T-AOC, and GSH in the intestinal contents of rainbow trout, while the level of SOD was significantly decreased, suggesting that peroxides may accumulate in the intestinal contents of rainbow trout under heat stress, which adversely affects the antioxidant capacity of the body. When subjected to environmental stress, the body will produce a large amount of superoxide radicals, which leads to the peroxidation of membrane lipids and the production of MDA. Excessive accumulation of MDA can cause cross-linking and polymerization of macromolecules such as proteins and nucleic acids, which can change the structure and function of cell membrane [8]. Therefore, membrane lipid peroxidation is an essential marker of cell membrane damage, and its content can directly reflect the level of plasma membrane peroxidation. GSH and SOD are crucial antioxidants that can remove excessive free radicals in the body and play a vital role in maintaining the normal physiological metabolic balance process. Many studies have shown the value of antioxidant supplements in alleviating the negative effects of heat stress in fish. Dietary supplementation of 5 mg/kg nanoselenium can effectively alleviate the tissue damage caused by heat stress in rainbow trout, and the activity of glutathione peroxidase in the liver was significantly increased, while the levels of alanine aminotransferase, aspartate aminotransferase, SOD, and MDA were decreased [8]. HSPs are one of the most vital biomarkers of heat stress response in animals [19]. In response to stress, the body can synthesize some endogenous protective proteins, such as HSPs, which play a protective and repair role in the heart, brain, liver, stomach, intestine, and other organs [8]. In the present study, the relative expression of *HSP90* and *HSP70* mRNA was significantly increased in the intestinal tissues of rainbow trout under heat stress. This result is consistent with the results of previous studies [8].

Heat stress activates the fish neuroendocrine function. The HPA and HSC axes were activated in fish under heat stress [5]. Exposure to high temperatures leads to HPA and HSC activation. For example, a rapid increase in plasma corticosterone concentration and a decrease in food intake can affect the integrity of intestinal epithelial cells [22]. In addition, the activation of the autonomic nervous system (ANS) under heat stress increases the level of pro-inflammatory cytokines and increases intestinal permeability, which also affects the homeostasis of the intestinal environment [23,24]. In the present study, HPA and HSC were activated in rainbow trout under heat stress, and the levels of hormones involved in stress regulation, including TRH, CRH, ACTH, MSH, β-END, TSH, T4, T3, cortisol, NE, Epi, and GH, were significantly increased. These results suggest that the neuroendocrine function is involved in the regulation of environmental adaptability in fish, and the increased activity of neuroendocrine function induced by heat stress may be related to the metabolic dysfunction.

### 3.2. The Barrier Structure of the Intestine Is Destroyed under Heat Stress

Many stressors, both acute and chronic, can disrupt intestinal barrier function in fish: for example, water dissolved oxygen, water temperature, diet composition, starvation, fasting, and infection [25]. It has been found that the intestinal barrier function of Atlantic salmon living in an adverse environment for a long time will be significantly reduced, which is a good experimental marker to evaluate the stress of fish [26]. In the present study, changes in intestinal permeability in rainbow trout under heat stress were first evaluated, and the levels of LPS and DAO in serum and intestinal contents were significantly increased, indicating increased intestinal permeability in rainbow trout.

At the molecular level, DEGs associated with intestinal permeability were significantly enriched in focal adhesion, regulation of actin cytoskeleton, tight junction, cellular senescence, apoptosis, adherens junction, peroxisome, and gap junction signaling pathways. These results indicate that the increased intestinal permeability in rainbow trout under heat stress is related to the activation of these signaling pathways. The intestinal barrier is a selective barrier formed by the interconnection of tight junction proteins between adjacent intestinal epithelial cells [18]. Under adverse environmental conditions, due to the occurrence of stress response, the tight junction between intestinal epithelial cells is destroyed, intestinal permeability is increased, and some harmful substances are infiltrated, leading to the occurrence of a systemic inflammatory response [14,23,27]. The effects of heat stress on fish intestinal epithelial cells may result from the reduction of blood supply to the intestine, which eventually leads to tissue ischemia and injury. In the present study, qRT-PCR results showed that the relative expression abundance of *ZO-1* and *claudin* mRNA in the intestine of rainbow trout was significantly increased under heat stress, while the relative expression abundance of *occludin* mRNA was significantly decreased. It was further confirmed that the increased intestinal permeability was related to the activation of signaling pathways related to intestinal permeability induced by heat stress.

Energy production in an organism is ultimately all related to mitochondria, and ambient temperature significantly affects mitochondrial efficiency and maximum capacity. The functional activity of mitochondria depends on oxygen as the ultimate electron acceptor, and thus cardiovascular function and oxygen transport link ambient temperature to energy metabolism. The growth efficiency of animals can change with the environmental temperature, and it has been shown that increased water temperature can lead to reduced growth and transformation efficiency of fish [2]. In addition, heat stress is able to disrupt cellular processes in animals, modulate metabolic responses, induce oxidative cell damage, and activate the apoptotic necrosis pathway. Thus, heat stress has been known to be cytotoxic [28,29]. Mitochondria are the major sites of ROS production and are the first to be damaged when the steady-state concentration of ROS changes. Related studies have shown that early pathological changes and mitochondrial morphological changes occur in skeletal muscle under heat stress [30]. Under acute heat stress, mitochondrial ROS production is increased in skeletal muscle, leading to oxidative damage of mitochondrial lipids and proteins [31]. Studies have shown that heat stress causes the overproduction of ROS and leads to the degeneration of mitochondrial proteins [32]. The cause of mitochondrial damage may be due to the lack of sufficient energy supply in cells under heat stress. In the present study, the morphological changes of intestinal and TJ-related organelles under heat stress were also confirmed by histopathology, SEM, and TEM. These results strongly suggest that heat stress leads to impaired intestinal barrier function and increased intestinal permeability in rainbow trout.

### 3.3. Heat Stress Promotes the Intestinal Inflammatory Response

The main effects of stress on the gut include changes in gastrointestinal motility, enhanced visceral perception, changes in gastrointestinal secretion, increased intestinal permeability, negative effects on gastrointestinal mucosal regeneration and mucosal blood flow, and negative effects on intestinal microbiota [17,33]. Mast cells are crucial effector cells of BGA, which can transform stress signals into a wide range of neurotransmitters and pro-inflammatory cytokines, and profoundly impact the physiology of the gastrointestinal tract [17]. It has been found that increased water temperature causes intestinal villus apoptosis and proximal intestinal edema in rainbow trout [27]. Our previous study also found that acute heat stress decreased the height of intestinal villi and epithelial cell thickness, while increased the thickness of the intestinal muscle layer and the number of goblet cells, accompanied by intestinal inflammation and intestinal mucosal injury in rainbow trout [6]. Histopathological examination is a technique that can reflect the normal and pathological conditions of fish. In the present study, significant changes in intestinal morphology and structure were observed in rainbow trout under heat stress. The intestinal mucosal epithelium of rainbow trout showed obvious degeneration, necrosis, and abscission under heat stress. There was red blood cell infiltration, inflammatory exudation, and edema in the lamina propria, submucosa, and muscle layer. The inflammation level of rainbow trout intestinal under heat stress was evaluated by a semi-quantitative scoring method, which further showed that there were obvious inflammatory responses and epithelial cell damage in rainbow trout intestine under heat stress. Therefore, heat stress caused obvious pathological damage in the intestine of rainbow trout.

The intestinal mucosa can synthesize and release a large number of mucoid substances, including lysozyme, macrophages, and other immune-active substances, which play an essential role in protecting the normal physiological activities of the host. When the water temperature increases, the environmental homeostasis in the gut of fish will be threatened. Some pathogenic microorganisms or toxins will penetrate into the mucosal immune barrier, thereby activating the innate or adaptive immune function of the body. Thereafter, the expression levels of several pro-inflammatory factors (such as IL-1β, IL-6, and TNF-α) in the gut and serum are significantly enhanced, thereby inducing the recruitment of innate immune cells to the inflammatory area [34]. In the present study, heat stress induced the activation of the TNF signaling pathway, TGF-β signaling pathway, MAPK signaling pathway, PPAR signaling pathway, Rap1 signaling pathway, and Ras signaling pathway, which may be the signaling pathways for the occurrence of intestinal injury and inflammatory response induced by heat stress in rainbow trout. Immune cytokines can reflect the physiological and pathological state. TNF is a pro-inflammatory cytokine that plays a vital role in animal immunity and cell homeostasis [35]. The TNF signaling pathway plays an essential role in the maintenance of tissue homeostasis, intestinal inflammation regulation, and apoptosis [36]. PPARs are ligand-activated transcription factors that play an essential role in fatty acid uptake, transport, and oxidation in mitochondria, peroxisomes, and microsomes, thereby inhibiting the secretion of pro-inflammatory cytokines [36]. In the present study, the levels of pro-inflammatory cytokines TNF-α, IL-6, and IL-1β in intestinal contents and serum were significantly increased, and the level of anti-inflammatory cytokine IL-10 was significantly decreased, indicating that the changes in inflammatory cytokines may be related to intestinal inflammatory response and intestinal mucosal injury. Previous studies have found that the expression of inflammatory factors (TNF-α and IL-8) in the proximal intestine of rainbow trout decreased after increasing water temperature [27]. The destruction of intestinal integrity causes LPS to enter the bloodstream, leading to the activation of the innate immune system and systemic inflammation [37]. In our previous study, the relative expression levels of intestinal tight junction proteins and pro-inflammatory cytokine-related genes were significantly decreased in rainbow trout under acute heat stress, suggesting that acute heat stress directly destroyed the intestinal barrier function of rainbow trout, thereby disrupting intestinal homeostasis [6]. Related studies have found that heat stress can reduce the diversity of intestinal microbiota and increase the expression level of intestinal cytokine genes in rainbow trout, but the addition of organic acids and nature-identical compounds in the diet did not reverse these effects [38]. Therefore, heat stress promoted the intestinal inflammatory response in rainbow trout.

### 3.4. Heat Stress Induces Dysbiosis of Intestinal Commensal Bacteria

The microbiota is an integral part of the intestinal barrier. The intestinal barrier is a crucial complex structure that serves as a boundary between the host and the environment and regulates interactions between bacteria and host cells [39]. In the present study, Proteobacteria were the most dominant bacteria in the intestinal microbiota of rainbow trout, and they were also the bacteria whose abundance was significantly increased under heat stress. The expansion of Proteobacteria is a microbial signature of intestinal dysbiosis [40]. It has been suggested that Proteobacteria are disruptors of intestinal homeostasis, and increased abundance of Proteobacteria is closely related to inflammatory bowel diseases (IBD) [40]. However, the exact mechanisms leading to an increase in Proteobacteria during the development of IBD are not fully understood. The prevailing consensus is the “oxygen hypothesis” that gut inflammation results in a decline in obligate anaerobes and an increase in facultative anaerobes (e.g., Enterobacteriaceae) [41]. Under normal physiological conditions, the intestinal epithelium is in a state of high oxygen consumption due to the oxidation and oxidative phosphorylation of fatty acids. The internal environment of the intestine is hypoxic, and the intestinal microflora dominated by obligate anaerobes is dominant [42]. This hypoxia state is of great significance for regulating intestinal barrier function and maintaining intestinal homeostasis. Heat stress can cause intestinal injury in rainbow trout, which promotes the abundance of Proteobacteria in the intestinal microbiota, especially the expansion of Enterobacteriaceae (facultative anaerobes), while the abundance of obligate anaerobes decreases, thereby promoting the dysbiosis of intestinal microbiota.

Bacteroidetes are one of the most common members of the intestinal microbiota and play a crucial regulatory role in the occurrence of intestinal diseases. A few Bacteroidetes are exogenous pathogens, while most are colonizers of the intestinal mucosa and coexist with the host and inhibit the colonization of intestinal pathogens [36]. In the present study, the abundance of Bacteroidetes and Firmicutes in the intestinal microbiota of rainbow trout were significantly decreased under heat stress. Bacteroidetes have been reported to be involved in carbohydrate transport and protein metabolism, which are important for the digestion of the diet [43]. Some studies have found that the toxicity of tartrazine affected the intestinal microbial community structure of *Crucian carp*, especially the number of *Bacteroides* and *Clostridium* decreased significantly [44]. These results suggest that the feeding, digestion, and nutrient transport of rainbow trout under heat stress may be affected by the decreased abundance of gut Bacteroidetes and Firmicutes. The ratio of Firmicutes to Bacteroidetes can reflect the physiological state of the gut, and a decrease in Firmicutes indicates intestinal dysfunction [36]. Firmicutes are considered to be the major butyrate-producing bacteria [45]. The microorganisms in Firmicutes can produce short-chain fatty acids (SCFAs), provide nutrition for intestinal mucosal cells, and help to maintain the normal function of the intestinal mucosa and regulate the intestinal micro-ecological environment [46,47]. Our previous study reported a significant reduction in the proportion of Firmicutes in the intestinal contents of rainbow trout under acute heat stress [6]. These results suggest that the intestinal commensal microbiota of rainbow trout is closely related to elevated water temperature, and the decreased abundance of intestinal Bacteroidetes and Firmicutes under heat stress affects the host’s environmental adaptability and the metabolic function of the gut.

### 3.5. Heat Stress Alters Gut Microbiota-related Metabolites

Heat stress alters lipid metabolism in rainbow trout. In the present study, some differential metabolites were involved in arachidonic acid metabolism, glycerophospholipid metabolism, and linoleic acid metabolism, which were all related to fatty acid metabolism. Arachidonic acid is the precursor of a variety of active substances, including prostaglandins, thromboxanes, and leukotrienes, and it plays an essential role in anti-inflammatory responses [48]. Previous studies have shown that prostaglandin I2 exerts anti-inflammatory effects by up-regulating cAMP and down-regulating NF-κB [49]. In the present study, the differential metabolites in the intestinal contents of rainbow trout, including leukotriene C4, prostaglandin I2, leukotriene E4, leukotriene A4, leukotriene D4, and PC, were all down-regulated under heat stress. The down-regulation of these differential metabolites indicated that the occurrence of intestinal inflammation in rainbow trout under heat stress was related to the activation of the arachidonic acid metabolic pathway. In the present study, the differential metabolites were significantly enriched in the glycerophospholipid metabolic pathway, and the differential metabolites phosphatidylcholine and LysoPC(18:1(11Z) were down-regulated, while choline and glycerophospholipid were up-regulated. LysoPC(18:1(11Z) belongs to the lysophosphatidylcholine (LPC) family. Studies have shown that LPCs are a major component of oxidized low-density lipoprotein and are considered pro-inflammatory factors [50,51]. Recent studies have shown that *Moringa oleiferis* polysaccharides may reduce intestinal inflammation by regulating the content of LPC [36]. Glycerophospholipid metabolism plays an essential role in stabilizing cell membranes. Glycerophospholipids (mainly phosphatidylethanolamine and phosphatidylcholine) are important components of cell membranes [52,53] and are closely related to cell membrane permeability and ATPase activity [54]. Regulation of glycerophospholipid content under heat stress may be an adaptive response to elevated temperature [55,56]. Serine, a modification group in the synthesis of phosphatidylserine (PtdSer), is an essential membrane phospholipid that can regulate the function and state of proteins in membranes and plays a vital role in cell metabolism [57]. The significant accumulation of serine with increasing temperature may help to regulate cell metabolism and maintain the structural stability of the cell membrane [58,59]. However, in the present study, the concentration of cholesterol in intestinal contents was significantly decreased under heat stress. Cholesterol is one of the main components of cell membrane, and it is also an important lipid in cell membrane. Cholesterol molecules are small and distributed in phospholipid bilayers. Cholesterol plays an essential role in regulating the fluidity of cell membrane, which is determined by its molecular structure. The polar head of the cholesterol molecule is the sterol ring hydroxyl group, close to the head of the phospholipid bilayer; the sterol ring is in the middle, and the tail is a long chain. Therefore, the contents of major phospholipids and cholesterol in the intestinal contents of rainbow trout were down-regulated under heat stress, indicating that heat stress affected the normal function of cell membrane.

It has been reported that a variety of differentially expressed metabolites have been identified in the liver of rainbow trout under heat stress, and these differential metabolites are mainly enriched in linoleic acid metabolism, α-linolenic acid metabolism, glycerophospholipid metabolism, and sphingolipid metabolism pathways [60]. In the present study, eight differential metabolites in the intestinal contents of rainbow trout under heat stress were significantly enriched in the linoleic acid metabolic pathway. Linoleic acid metabolism and α-linolenic acid metabolism are two basic types of polyunsaturated fatty acid (PUFAs) metabolism, and their metabolic changes are often used to identify oxidative stress. In the present study, propionic acid metabolism was enriched to two differential metabolites, and both lactate and propionyl phosphates were significantly down-regulated. Lactate, an intermediate of glycolysis, can regulate the immune response and intestinal mucosal tissue regeneration [61]. Lactate was reported to be negatively correlated with the relative abundance values of *Romboutsia*, *Methylowarbia*, *Desulfobacca*, and *Methylospora* [62]. The gut is essential for glucose absorption, and inadequate glucose absorption ultimately affects fish growth [62]. Therefore, it is crucial to determine the interactions between key nutrients and specific microorganisms for the intervention of intestinal post-injury repair under heat stress.

Previous studies have shown that bile acids are vital regulators of intestinal epithelial barrier function. Mechanistically, bile acids inhibit the activation of PPARα, leading to the blocking of fatty acid oxidation and the regeneration of damaged intestinal stem cells [63]. In the present study, the differential metabolites were significantly enriched in the bile acid secretion pathway and the primary bile acid biosynthesis pathway, and the significant down-regulation of metabolite cholesterol was negatively correlated with the abundance of Parasutterella in gut microbiota. Studies have shown that *Rhodiola* extract plays a crucial role in preventing intestinal microbiota imbalance and intestinal inflammation by restoring the richness and diversity of intestinal microorganisms, reducing the abundance of Proteobacteria and the conditional pathogenic bacteria *Parasutterella* and *Staphylococcus* and increasing the abundance of beneficial microorganisms in *Lactobacillus* and *Bifidobacterium* [64].

Heat stress alters amino acid metabolism. Amino acids are the basic functional units of protein molecules and one of the most essential nutrients for organisms [36]. In the present study, the differential metabolites were involved in protein digestion and absorption, biosynthesis of amino acids, and aminoacyl-tRNA biosynthesis. Previous studies have shown that amino acid metabolism disorders can lead to immune disorders and oxidative stress, ultimately resulting in arrested muscle growth and weight loss [65,66]. Aminoacyl-tRNA plays an essential role in inflammation, immune response, tumorigenesis, and other physiological or pathological processes [66,67], suggesting that the intestinal injury and inflammatory response caused by heat stress in rainbow trout may be related to the abnormal biosynthesis of aminoacyl-tRNA. The abundance and composition of gut microbiota also affect the digestion and absorption of amino acids by the host [68]. Under heat stress, the intestinal differential metabolites of rainbow trout were mainly enriched in the pathways related to amino acid metabolism, especially protein digestion and absorption pathways and amino acid biosynthesis pathways. For example, *Clostridium* is a key driver of amino acid fermentation, while *Peptostreptococcus* is a key driver of glutamate or tryptophan utilization [69].

### 3.6. Heat Stress Induces Intestinal Injury by Activating PPAR-α Signaling Pathway

PPAR is a member of the ligand-activated nuclear transcription factor superfamily, including PPAR-α, PPAR-β/δ, and PPAR-γ phenotypes [70]. In the present study, a total of 73 DEGs were enriched in the PPAR signaling pathway. The activation of the PPAR pathway is usually related to lipid metabolism. PPAR-α regulates cholesterol metabolism by down-regulating the target gene *CYP8B1* through the adipokine signaling pathway. It affects lipogenesis by up-regulating the target gene *SCD-1*. It also affected the process of lipid transport by down-regulating the target genes *Apo-AⅠ* and *Apo-AⅡ*. These results indicated that the fatty acid production of rainbow trout was enhanced, while the cholesterol metabolism and lipid transport ability were decreased under heat stress. It has been suggested that the internal environment of the gut is hypoxic, and the intestinal microbial microbiota dominated by obligate anaerobes is dominant [42]. This state of hypoxia is of great significance for regulating intestinal barrier function and maintaining intestinal homeostasis. Under normal conditions, the PPAR-γ signaling pathway activated by butyrate in the gut polarizes intracellular metabolism into mitochondrial β-oxidation of fatty acids, thereby increasing oxygen consumption of intestinal epithelial cells and promoting hypoxia of the intestinal epithelium [71,72]. Thus, intestinal epithelial hypoxia ensures the production of SCFAs by obligate anaerobes, which in turn induces intestinal epithelial hypoxia, thereby maintaining intestinal homeostasis and a virtuous cycle of intestinal microbial community composition [71]. Activation of PPAR-γ signaling by the gut microbiota is a homeostatic pathway that inhibits the expansion of potentially pathogenic bacteria *Escherichia* and *Salmonella* due to dysregulation of gut homeostasis [71].

Bile acids are the final product of cholesterol breakdown in the liver. They regulate energy metabolism and immune function mainly through farnesoid X receptor [63]. Previous studies have shown that bile acids are important regulators of intestinal epithelial barrier function, but the underlying mechanism remains unclear. In the present study, the activation of the PPAR-α signaling pathway in the intestine of rainbow trout under heat stress affected the body cholesterol metabolism by down-regulating the target gene *CYP8B1* through the adipocytokine signaling pathway. Studies have found that patients with IBD and mice with colitis have a disorder of bile acid metabolism, and the level of cholic acid (CA) is significantly up-regulated. In addition, *CYP8B1*, a metabolic enzyme in the classical bile acid synthesis pathway of the liver, was over-activated [63]. Exogenous CA supplementation or overexpression of *CYP8B1* can aggravate the IBD phenotype and damage the intestinal barrier and its repair function in mice, while interference with *CYP8B1* expression can promote the relief of intestinal inflammation and restore the ability of intestinal epithelial regeneration [63]. *SCD1* is a rate-limiting enzyme required to convert long-chain saturated fatty acids into long-chain monounsaturated fatty acids. *SCD1* can convert the fat in stearic acid to oleic acid. In the present study, the target gene *SCD1* was upregulated, and body fat tended to be unsaturated. Cell membranes are composed of fatty acids; some of them can be saturated, but most of them require unsaturated fatty acids for gaining greater fluidity. Under heat stress, the fatty acid production process of rainbow trout is enhanced, while the cholesterol metabolism and lipid transport ability are decreased. Therefore, these results suggest that heat stress promotes the intestinal injury of rainbow trout by inducing the activation of the PPAR-α signaling pathway to enhance the production of fatty acids and reduce cholesterol metabolism and lipid transport ability.

## 4. Materials and Methods

### 4.1. Fish and Facilities

The study was carried out in the Water Circulation Aquaculture System of Linxia Salmon-Trout Breeding Center (35°60′ N 103°21′ E; Linxia, China). Full-sib Norwegian rainbow trout with similar body length and average body weight of 182.4 ± 5.1 g (mean ± SD) were selected as the experimental material and were domesticated at 16 °C water temperature for 2 weeks. The study was divided into control (CO) and heat stress (HS) groups, with three biological replicates per group and 20 fish under each replicate. A total of 120 fish were evenly distributed among six tanks. This study was completed in a water cycle aquaculture system, using the methods of physical filtration and biological filtration to achieve the purification and reuse of water. The Water Quality Monitoring System (YOSEMITE TECHNOLOGIES Co., Ltd., Suzhou, China) can measure multiple indicators of circulating water in real-time, including pH, dissolved oxygen, temperature, ammonia nitrogen, nitrite, nitrate, total phosphorus, and total nitrogen. Fish were fed twice per day during the experiment, at 8:00 AM and 5:00 PM, respectively. The pellet feed was purchased from Beijing Han-Ye Technology Co., Ltd. (Beijing, China; crude protein ≥ 48.0%, crude fat ≥ 10.0%). At the beginning of the experiment, the optimal growth temperature of rainbow trout was set at 16 °C as the control condition. The maximum tolerated temperature of rainbow trout was set at 24 °C as the stress temperature, and the stress lasted for 21 days. Each 600 L cylindrical cement tank (100 cm in diameter) was equipped with three sets of electric heating rods (1000 W each) and automatic temperature control to monitor the water temperature continuously. The water temperature gradually increased from 16 °C to 24 °C, with an average increase of 1 °C per day for 8 days. The water cycle rate was adjusted to a minimum level and had almost no effect on the water temperature. Three aeration pumps (each 45 W) were used for continuous oxygen supply to increase the dissolved oxygen concentration in the water, and the dissolved oxygen content was not less than 7 mg/L. Other experimental conditions were consistent with our published paper [6]. This study was conducted in strict accordance with the requirements of the animal Ethics Committee and approved by the Ethics Committee of the School of Life Sciences, Lanzhou University (Approval number: EAF2021042).

### 4.2. Sample Collection

After the experiment, the fish were quickly anesthetized with 200 mg/L tricaine methanesulfonate (MS-222; Argent Chemical Laboratories, Redmond, WA, USA). Blood samples were collected from the caudal vein using a vacuum blood collection tube containing ethylenediamine tetraacetic acid (EDTA) anticoagulant and sterilized blood collection needle. The serum was obtained by centrifugation at 1000 rpm/min for 10 min. Later, each fish was dissected from the ventral side using sterile instruments on an ultra-clean workbench. Three fish were removed from each tank, cut at the distal intestine (0.5 cm from the anus), and the intestinal contents of the same group (9 fish) were squeezed into a shared sterile tube, mixed, and dispensed in six different sterile Eppendorf tubes. Before the intestinal mucosa samples were extracted, the intestinal contents were rinsed with phosphate-buffered saline (PBS). The intestinal mucosa was scraped with a surgical blade and transferred into sterile Eppendorf tubes for freezing. Finally, the proximal gut, midgut, and distal gut of fish were fixed in 4% paraformaldehyde and 2.5% glutaraldehyde solution for the paraffin section and electron microscope sample preparation.

### 4.3. Histology

The gut tissue of rainbow trout was fixed with 4% paraformaldehyde, dehydrated with ethanol, equilibrated in xylene, and embedded in paraffin. Tissue sections were cut (longitudinal cuts of ~5 μm) using a tissue slicer (Leica, CM3050S, Wetzlar, Germany), stained with hematoxylin and eosin (H&E), examined under a light microscope, and analyzed with ImageJ 1.5v image analysis software.

Prefixed with 3% glutaraldehyde, the tissue was then post-fixed in 1% osmium tetroxide, dehydrated in series acetone, infiltrated in Epox 812 for somewhat longer, and embedded. The semithin sections were stained with methylene blue, and the ultrathin sections were cut with a diamond knife and stained with uranyl acetate and lead citrate. Sections were examined with a JEM-1400-Flash Transmission Electron Microscope (TEM).

The scanning electron microscopy (SEM) procedure was as follows: the fixed sample was washed twice with PBS for 5 min each time and dehydrated with serial gradients of alcohol at 30%, 50%, 70%, 80%, 90%, 95%, and 100% for 10 min with each gradient. The samples were gently fixed with conductive glue, critical point dried (Critical Point Drying, K850, Quorum, UK), sprayed by ion sputtering, and finally observed at the appropriate position and appropriate fold under the microscope (SEM, Inspect, FEI, Hillsboro, OR, USA).

### 4.4. Gut Inflammation, Gut Permeability, and Stress Hormone

Serum and intestinal contents were examined for inflammatory cytokines, intestinal permeability, and stress markers. The Enzyme-Linked Immunosorbent Assay (ELISA) Kit was provided by Meimian Industrial Co., Ltd. (Yancheng, China). The serum and gut contents were analyzed for superoxide dismutase (SOD), malonaldehyde (MDA), glutathione (GSH), total antioxidation capacity (T-AOC), diamine oxidase (DAO), lipopolysaccharide (LPS), interleukin 1β (IL-1β), interleukin 6 (IL-6), interleukin 10 (IL-10), tumor necrosis factor-α (TNF-α), and transforming growth factor α (TFG-α). The serum was analyzed for thyrotropin-releasing hormone (TRH), corticotropin-releasing hormone (CRH), adrenocorticotropic hormone (ACTH), melanocyte-stimulating hormone (MSH), β-endorphin (β-END), thyroid stimulating hormone (TSH), thyroid hormone (T4), thyroid hormone (T3), cortisol, norepinephrine (NE), epinephrine (Epi), and growth hormone (GH). The absorbance was measured with an RT-6100 microplate reader (Rayto, Shanghai, China), and the concentration in each sample was calculated according to the standard curve.

### 4.5. Analysis of the Gut Microbiota

The intestinal contents of 200 mg (per sample) were transferred to sterile tubes containing 0.5 g of 0.1 mm silica beads and homogenized in a Precellys homogenizer (Bertin Instruments, Montigny-le-Bretonneux, France) for two cycles of 60 s at 6000 rpm/min followed by 5 min rest on ice. DNA was extracted from the intestinal contents using a QIAamp Fast DNA Stool Mini Kit (Qiagen Gmbh, Hilden, Germany) according to product instructions. The concentration and purity of DNA were determined using a Nanodrop 2000 (Thermo Fisher Scientific, Waltham, MA, USA). DNA integrity was determined by 1% agar-gel electrophoresis. The V3–V4 regions of 16S rRNA were amplified by PCR using the forward primer 338F (5′-ACTCCTACGGGAGGCAGCA-3′) and the reverse primer 806R (5′-GGACTACHVGGGTWTCTAAT-3′). The PCR amplification process and product purification were performed according to our published papers [6].

The library preparation and sequencing work was completed on an Illumina HiSeq 2500 platform (Beijing Baimaike Biotechnology Co., Ltd., Beijing, China). Data analysis was performed using BMKCloud (www.biocloud.net, accessed on 14 January 2023). Data preprocessing included quality filtering, double-ended sequence splicing, and removal of chimeras. The raw reads obtained by sequencing were filtered using Trimmomatic (version 0.33) software. Then, Cutadapt (version 1.9.1) software was used to identify and remove the primer sequence, and clean reads without primer sequence were obtained. The dual-end reads were spliced by Usearch (version 10) software, and the chimera was removed by Uchime (version 8.1) software to obtain effective reads. The reads with 97.0% similarity were clustered using Usearch (Version 10.0) software, and the threshold was set to 0.005% for filtering to obtain operational taxonomic units (OTUs). Using Silva (Release132, http://www.arb-silva.de, accessed on 14 January 2023) as the reference database, the naive Bayes classifier was used to make a taxonomic annotation for the feature sequences; then, the species classification information corresponding to each feature was obtained, and later the community composition of each sample was counted at all levels (phylum, class, order, family, genus, species). Information analysis included OTU division, diversity analysis, difference analysis, and correlation analysis.

QIIME2 (https://qiime2.org/, accessed on 14 January 2023) was used for bioinformatics analysis of the obtained data. Wilcoxon rank-sum test was used to analyze the alpha diversity of intestinal microorganisms including ACE, Chao1, Shannon, and Simpson indices. The Bray–Curtis algorithm was used to evaluate the beta diversity of all samples, including unweighted Unifrac distance-based principal coordinate analysis (PCoA) and binary Jaccard-based non-metric multidimensional scaling (NMDS) analysis. The permutational multivariate analysis of variance (PERMANOVA) test was used to assess significant differences among groups. Linear effect size feature selection (LEfSe) analysis (http://huttenhower.sph.harvard.edu/lefse/, accessed on 14 January 2023) was used to evaluate significant differences among groups, using species with significant differences as biomarkers. Wilcoxon rank-sum test was used for difference analysis, and linear discriminant analysis (LDA) was used to estimate the effect of the degree of these bacteria (from phylum to genus level). We identified the bacteria with an LDA score of ≥4 as “discriminative bacteria”.

### 4.6. Analysis of Intestinal Metabolic Profile

Ultra-high performance liquid chromatography-mass spectrometry (UPLC-MC) technology was adopted to analyze the metabolic profiles in the CO and HS groups. A thawed sample (100 mg) was accurately weighed and promptly ground into powder. The sample was homogenized in 1 mL precooled methanol/acetonitrile/ddH_2_O solvent (2:2:1, *v*/*v*/*v*). The mixture was treated with cryogenic ultrasound at −20 °C for 30 min and then centrifuged at 4 °C and 14,000 rpm for 15 min. The supernatant was extracted and lyophilized by a vacuum lyophilizer, and then the dried sample was redissolved in 100 μL acetonitrile/ddH_2_O solvent (1:1, *v*/*v*) for homogenization. After centrifugation at 4 °C and 14,000 rpm for 15 min, the supernatant was collected for subsequent analyses. Additional quality control (QC) samples were prepared to monitor the repeatability and stability of the instrument.

The Agilent 1290 Infinity LC UHPLC system was adopted for liquid chromatography, and the parameters were set as follows: flow velocity, 0.5 mL/min; column temperature, 25 °C; injection volume, 2 μL; mobile phase A, 25 mM ammonium hydroxide + 25 mM ammonium acetate; mobile phase B, acetonitrile. The elution procedure was as follows: 0–0.5 min, a maintained at 5%; 0.5–7 min, a changed from 5 to 35% linearly; 7–8 min, a changed from 35 to 60% linearly; 8–9 min, a was 60%; 9–9.1 min, a linearly changed from 60 to 5%; 9.1–12 min, a maintained at 5%. The AB Sciex Triple TOF 6600 mass spectrometer was selected in the present study, and the parameters were set up in accordance with the study reported by Tian et al. [73]. For the electrospray ionization source: ion source gas 1, 60 kPa; ion source gas 2, 60 kPa; curtain gas, 30 kPa; ion spray voltage, ±5500 V; source temperature, 600 °C. For MS-only acquisition, TOF MS scan m/z range: 60–1000 Da; product ion scan m/z range: 25–1000 Da; TOF MS scan accumulation time: 0.20 s/spectra; product ion scan accumulation time: 0.05 s/spectra.

After the analyses were conducted, the raw data in the wiff.scan format were converted into MzXML format by ProteoWizard MSConvert software (Version 3.0) and then imported into XCMS software (Version 3.2). CAMERA software (Version 4.3) was adopted for the annotation of isotopes and adducts. Subsequently, PCA, projections to latent structure-discriminant analysis (PLS-DA) and orthogonal PLS-DA (OPLS-DA) were conducted by SIMCA-P 14.1 software, and the variable importance in projection (VIP) of each metabolite was calculated. Metabolites with VIP > 1, *p* < 0.05 and fold change (FC) > 1.5 or <0.667 were considered as differential metabolites. The functional annotation and enrichment analysis of differential metabolites were conducted using the Kyoto Encyclopedia of Genes and Genomes (KEGG) database. Furthermore, correlation analyses between differential bacteria and metabolites were performed, and Pearson correlation coefficient (CC) was selected. *p* < 0.05 and CC > 0.8 or <−0.8 were considered relevant.

### 4.7. Transcriptome Sequencing and Analysis

The gut samples in the CO and HS groups were selected for transcriptome analysis. Total RNA was extracted from the intestinal mucosa of each sample using a mirVana™ miRNA Isolation Kit (Ambion, Austin, TX, USA) following the manufacturer’s instructions. The concentration, quality, and integrity of the RNA samples were determined by NanoDrop2000 spectrophotometer (Thermo, Waltham, MA, USA). RNA with 28S/18S ≥ 1.5 and RNA integrity number >8.0 were selected for further analysis. The ribosomal RNA (rRNA) in total RNA was removed using the Ribo-Zero™ rRNA Removal Kit. A single strand of complementary DNA (cDNA) was synthesized using the TruSeq^®^ Stranded Kit containing random primers and reverse transcriptase. The double-stranded cDNA was synthesized using DNA polymerase I and ribonuclease H (RNaseH); the double-stranded cDNA product was amplified and purified to obtain the cDNA library. High-throughput sequencing was completed using the Illumina HiSeq 2500 platform (Beijing Baimaike Biotechnology Co., Ltd.) After sequencing was conducted, Cutadapt software (version 1.18) was used to filter the original data to obtain the effective sequence. FastQC online tool was used to calculate the Q20 and Q30 contents and evaluate the quality of the sequencing data. The proportion of Q30 > 85% indicated high quality, and the subsequent analysis was based on high-quality sequencing data. HISAT2 software (Version 2.0.5) was selected to compare the filtered sequences to the reference genome, HTSeq software (Version 2.0) was used to calculate the read counts of each gene, the expression of each gene was quantified as transcripts per million reads (TPM), and edge R package in R software (Version 4.0.5) was used to analyze differentially expressed genes (DEGs).

The concentration of candidate genes in different functional modules was determined by the gene ontology (GO: http://www.geneontology.org; accessed on 14 January 2023) and KEGG (http://www.genome.jp/kegg/pathway.html; accessed on 14 January 2023) enrichment analysis. If candidate genes were significantly enriched in a functional module, the proportion of candidate genes in the functional module was increased considerably. Each functional module had a *p*-value, and the smaller the *p*-value, the richer the candidate genes were in this functional module. Subsequently, the false discovery rate (FDR) correction for *p*-value, the function of FDR ≤ 0.01, was set as a significant enrichment.

### 4.8. Quantitative Real-Time PCR

The gut samples from the CO and HS groups were used to extract total RNA with TRIzol™ reagent (Invitrogen, California, USA). The Prime ScriptTM RT reagent Kit with gDNA Eraser (Takara, Dalian, China) was used to synthesize the first-strand cDNA. To remove genomic DNA: 5 × gDNA Eraser Buffer 2.0 µL, gDNA Eraser 1.0 µL, total RNA (1 pg–500 ng) 1.0 µL, add RNasefree ddH_2_O to 10.0 µL, instantaneous centrifuge, reaction at 42 °C for 2 min. The reaction system was as follows: total RNA 1.0 µL, gDNA Eraser 1.0 µL, 5 × gDNA Eraser Buffer 2.0 µL, RNase-free water 6.0 µL. The cycling parameters were as follows: 42 °C for 15 min, and 85 °C for 5 s. Gene-specific primers were designed using Oligo 7 according to each identified gene sequence of the intestinal mucosa transcriptome library. All primers used were synthesized by Sangong Biotech Co., Ltd. (Shanghai, China). The list of primers is shown in Supplementary Table S1. The expression of each sample was checked in triplicate using the PrimeScript™ RT Master Mix (Perfect Real-Time) in 20 μL reaction volumes, according to the manufacturer’s instructions. The reaction system was as follows: 2 × QuantiFast SYBR Green RT-PCR Master Mix 10.0 µL; RNase-free water 6.2 µL; template RNA 1.0 µL; forward prime 1.4 µL; reverse prime 1.4 µL. Reaction process: pre-denaturation at 95 °C for 15 min; denaturation at 95 °C for 10 s; annealing at 56.0 °C for 45 s; 95 °C extension, 15 s; 60 °C, 1 min; 95 °C extension, 15 s; 40 cycles; 4 °C, stored. The *β-actin* was used as an internal reference and amplified using ABI 7500 quantitative real-time PCR (qRT-PCR). Disassociation curve analysis was performed to check amplification specificity. Each relative expression result was calculated using the 2^−ΔΔCT^ method.

### 4.9. Data Statistics and Analysis

All data were presented as the mean ± standard deviation (SD). Statistical analysis of the difference test was performed by one-way analysis of variance (ANOVA) using SPSS 22.0 and GraphPad Prism 7 software. *, *p* < 0.05; **, *p* < 0.01; ***, *p* < 0.001 using a one-way analysis of variance (ANOVA) followed by Tukey’s HSD test.

## 5. Conclusions

Conclusively, this study is the first to investigate the mechanism of heat stress-induced intestinal injury in rainbow trout by combined analysis of gut physiology and biochemistry, intestinal histology, gut microbiota, and gut metabolites under heat stress. Oxidative stress occurs in the intestine under heat stress, which leads to an increase in intestinal permeability and the occurrence of inflammatory response, thereby destroying the structure and function of the intestinal barrier. Heat stress alters the composition and abundance of the gut microbiota. At the phylum level, the abundance of Proteobacteria increased, while that of Firmicutes and Bacteroidetes decreased. At the genus level, the abundance of *Enterobacteriaceae* and *Lactobacillus* increased, whereas that of *Clostridium*, *Acinetobacter*, and *Bacillus* decreased. The differential metabolites in the intestinal contents of rainbow trout under heat stress were significantly enriched in glycerophospholipid metabolism, choline metabolism, linoleic acid metabolism, taurine and hypotaurine metabolism, bile secretion, and arachidonic acid metabolism signaling pathways. Finally, transcriptome sequencing of intestinal mucosa revealed that heat stress promoted intestinal injury in rainbow trout by inducing activation of the PPAR-α signaling pathway, enhancing fatty acid production, and reducing cholesterol metabolism and lipid transport capacity.

## Figures and Tables

**Table 1:** Pathological injury scores of intestinal tissue under acute heat stress.

	N = 8	Lamina Propria Inflammation		Villus Damage		Edema	
		Mean	SD	Mean	SD	Mean	SD
Proximal gut	CO	1.16 ^a^	0.14	0.63 ^a^	0.26	0.30 ^a^	0.12
	HS	3.65 ^b^	0.42	4.04 ^b^	0.48	4.38 ^b^	0.27
Mid gut	CO	1.47 ^a^	0.27	0.95 ^a^	0.29	0.30 ^a^	0.10
	HS	4.08 ^b^	0.35	3.19 ^b^	0.25	3.93 ^b^	0.21
Distal gut	CO	1.13 ^a^	0.08	0.45 ^a^	0.17	1.15 ^a^	0.18
	HS	3.51 ^b^	0.32	3.15 ^b^	0.28	3.30 ^b^	0.41

A score of 1–2 represents normal morphology, a score of 3–4 indicates distinct morphological signs of inflammation, while a score of 5 represents severe symptoms of enteritis. Means ± SD; different letters (a, b) indicate a statistically significant difference among groups at significance level *p* < 0.05. CO, control groups; HS, heat stress groups.

## Data Availability

The original contributions presented in the study are publicly available. This data can be found here: https://doi.org/10.6084/m9.figshare.22793705 (accessed on 1 March 2023).

## Supplementary Materials

The following supporting information can be downloaded at: https://www.mdpi.com/article/10.3390/ijms24108569/s1.

## References

[1] Alfonso S., Gesto M., Sadoul B. (2020). Temperature increase and its effects on fish stress physiology in the context of global warming. J. Fish Biol..

[2] Little A.G., Loughland I., Seebacher F. (2020). What do warming waters mean for fish physiology and fisheries?. J. Fish Biol..

[3] Zhou C.-Q., Zhou P., Ren Y.-L., Cao L.-H., Wang J.-L. (2019). Physiological response and miRNA-mRNA interaction analysis in the head kidney of rainbow trout exposed to acute heat stress. J. Therm. Biol..

[4] Huyben D., Sun L., Moccia R., Kiessling A., Dicksved J., Lundh T. (2018). Dietary live yeast and increased water temperature influence the gut microbiota of rainbow trout. J. Appl. Microbiol..

[5] Bonga S.E.W. (1997). The stress response in fish. Physiol. Rev..

[6] Zhou C., Yang S., Ka W., Gao P., Li Y., Long R., Wang J. (2022). Association of Gut Microbiota With Metabolism in Rainbow Trout Under Acute Heat Stress. Front. Microbiol..

[7] Nardocci G., Navarro C., Cortés P.P., Imarai M., Montoya M., Valenzuela B., Jara P., Acuña-Castillo C., Fernández R. (2014). Neuroendocrine mechanisms for immune system regulation during stress in fish. Fish Shellfish. Immunol..

[8] Li L., Liu Z., Quan J., Lu J., Zhao G., Sun J. (2022). Dietary nanoselenium supplementation for heat-stressed rainbow trout: Effects on organizational structure, lipid changes, and biochemical parameters as well as heat-shock-protein- and selenoprotein-related gene expression. Fish Physiol. Biochem..

[9] Zhu L., Zhu W., Zhao T., Chen H., Zhao C., Xu L., Chang Q., Jiang J. (2021). Environmental Temperatures Affect the Gastrointestinal Microbes of the Chinese Giant Salamander. Front. Microbiol..

[10] Fontaine S.S., Novarro A.J., Kohl K.D. (2018). Environmental temperature alters the digestive performance and gut microbiota of a terrestrial amphibian. J. Exp. Biol..

[11] Bestion E., Jacob S., Zinger L., Di Gesu L., Richard M., White J., Cote J. (2017). Climate warming reduces gut microbiota diversity in a vertebrate ectotherm. Nat. Ecol. Evol..

[12] Zhu L., Liao R., Wu N., Zhu G., Yang C. (2018). Heat stress mediates changes in fecal microbiome and functional pathways of laying hens. Appl. Microbiol. Biotechnol..

[13] Pennisi E. (2020). Living with heat. Science.

[14] Koch F., Thom U., Albrecht E., Weikard R., Nolte W., Kuhla B., Kuehn C. (2019). Heat stress directly impairs gut integrity and recruits distinct immune cell populations into the bovine intestine. Proc. Natl. Acad. Sci. USA.

[15] Cao C., Chowdhury V.S., Cline M.A., Gilbert E.R. (2021). The Microbiota-Gut-Brain Axis during Heat Stress in Chickens: A Review. Front. Physiol..

[16] Pearce S.C., Mani V., Weber T.E., Rhoads R., Patience J.F., Baumgard L.H., Gabler N.K. (2013). Heat stress and reduced plane of nutrition decreases intestinal integrity and function in pigs1. J. Anim. Sci..

[17] Konturek P.C., Brzozowski T., Konturek S.J. (2011). Stress and the gut: Pathophysiology, clinical consequences, diagnostic approach and treatment options. J. Physiol. Pharmacol..

[18] Rostagno M.H. (2020). Effects of heat stress on the gut health of poultry. J. Anim. Sci..

[19] Varasteh S., Braber S., Akbari P., Garssen J., Fink-Gremmels J. (2015). Differences in Susceptibility to Heat Stress along the Chicken Intestine and the Protective Effects of Galacto-Oligosaccharides. PLoS ONE.

[20] Turnbaugh P., Gordon J.I. (2008). An Invitation to the Marriage of Metagenomics and Metabolomics. Cell.

[21] Miller M.R., Nichols P.D., Barnes J., Davies N.W., Peacock E.J., Carter C.G. (2006). Regiospecificity profiles of storage and membrane lipids from the gill and muscle tissue of atlantic salmon (*Salmo salar* L.) grown at elevated temperature. Lipids.

[22] Lambert G.P. (2009). Stress-induced gastrointestinal barrier dysfunction and its inflammatory effects1. J. Anim. Sci..

[23] Chelakkot C., Ghim J., Ryu S.H. (2018). Mechanisms regulating intestinal barrier integrity and its pathological implications. Exp. Mol. Med..

[24] Ulrich-Lai Y., Herman J. (2009). Neural regulation of endocrine and autonomic stress responses. Nat. Rev. Neurosci..

[25] Jutfelt F.J.E. (2011). Barrier function of the gut. Encycl. Fish Physiol. Genome Environ..

[26] Sundh H., Kvamme B.O., Fridell F., Olsen R.E., Ellis T., Taranger G.L., Sundell K. (2010). Intestinal barrier function of Atlantic salmon (*Salmo salar* L.) post smolts is reduced by common sea cage environments and suggested as a possible physiological welfare indicator. BMC Physiol..

[27] Huyben D., Vidakovic A., Sundh H., Sundell K., Kiessling A., Lundh T. (2019). Haematological and intestinal health parameters of rainbow trout are influenced by dietary live yeast and increased water temperature. Fish Shellfish. Immunol..

[28] Du J., Di H.-S., Guo L., Li Z.-H., Wang G.-L. (2008). Hyperthermia causes bovine mammary epithelial cell death by a mitochondrial-induced pathway. J. Therm. Biol..

[29] Davidson J.F., Schiestl R.H. (2001). Cytotoxic and Genotoxic Consequences of Heat Stress Are Dependent on the Presence of Oxygen in *Saccharomyces cerevisiae*. J. Bacteriol..

[30] Hsu Y.D., Chen S.S., Lee W.H., Lin S.Z., Kao M.C., Tsao W.L. (1995). Mitochondrial alterations of skeletal muscle in a heat stress rat model. Proc. Natl. Sci. Counc. Repub. China Part B Life Sci..

[31] Mujahid A., Pumford N.R., Bottje W., Nakagawa K., Miyazawa T., Akiba Y., Toyomizu M. (2007). Mitochondrial oxidative damage in chicken skeletal muscle induced by acute heat stress. J. Poult. Sci..

[32] Belhadj Slimen I., Najar T., Ghram A., Dabbebi H., Ben Mrad M., Abdrabbah M. (2014). Reactive oxygen species, heat stress and oxidative-induced mitochondrial damage. A review. Int. J. Hyperth..

[33] McEwen B.S., Gianaros P.J. (2011). Stress- and Allostasis-Induced Brain Plasticity. Annu. Rev. Med..

[34] Yu Y., Ding L., Huang Z., Xu H., Xu Z. (2021). Commensal bacteria-immunity crosstalk shapes mucosal homeostasis in teleost fish. Rev. Aquac..

[35] Brenner D., Blaser H., Mak T.W. (2015). Regulation of tumour necrosis factor signalling: Live or let die. Nat. Rev. Immunol..

[36] Tian H., Wen Z., Liu Z., Guo Y., Liu G., Sun B. (2022). Comprehensive analysis of microbiome, metabolome and transcriptome revealed the mechanisms of Moringa oleifera polysaccharide on preventing ulcerative colitis. Int. J. Biol. Macromol..

[37] Hall D.M., Buettner G.R., Oberley L.W., Xu L., Matthes R.D., Gisolfi C.V., Laitano O., Leon L.R., O Roberts W., Sawka M.N. (2001). Mechanisms of circulatory and intestinal barrier dysfunction during whole body hyperthermia. Am. J. Physiol. Circ. Physiol..

[38] Pelusio N.F., Rossi B., Parma L., Volpe E., Ciulli S., Piva A., D’Amico F., Scicchitano D., Candela M., Gatta P.P. (2020). Effects of increasing dietary level of organic acids and nature-identical compounds on growth, intestinal cytokine gene expression and gut microbiota of rainbow trout (*Oncorhynchus mykiss*) reared at normal and high temperature. Fish Shellfish. Immunol..

[39] Scaldaferri F., Pizzoferrato M., Gerardi V., Lopetuso L., Gasbarrini A. (2012). The gut barrier: New acquisitions and therapeutic approaches. J. Clin. Gastroenterol..

[40] Rizzatti G., Lopetuso L.R., Gibiino G., Binda C., Gasbarrini A. (2017). Proteobacteria: A Common Factor in Human Diseases. BioMed Res. Int..

[41] Rigottier-Gois L. (2013). Dysbiosis in inflammatory bowel diseases: The oxygen hypothesis. ISME J..

[42] Litvak Y., Byndloss M.X., Bäumler A.J. (2018). Colonocyte metabolism shapes the gut microbiota. Science.

[43] Karlsson F.H., Ussery D.W., Nielsen J., Nookaew I. (2011). A Closer Look at Bacteroides: Phylogenetic Relationship and Genomic Implications of a Life in the Human Gut. Microb. Ecol..

[44] Wu L., Xu Y., Lv X., Chang X., Ma X., Tian X., Shi X., Li X., Kong X. (2021). Impacts of an azo food dye tartrazine uptake on intestinal barrier, oxidative stress, inflammatory response and intestinal microbiome in crucian carp (*Carassius auratus*). Ecotoxicol. Environ. Saf..

[45] Parada Venegas D., De La Fuente M.K., Landskron G., González M.J., Quera R., Dijkstra G., Harmsen H.J.M., Faber K.N., Hermoso M.A. (2019). Short Chain Fatty Acids (SCFAs)-Mediated Gut Epithelial and Immune Regulation and Its Relevance for Inflammatory Bowel Diseases. Front. Immunol..

[46] Koh A., De Vadder F., Kovatcheva-Datchary P., Bäckhed F. (2016). From Dietary Fiber to Host Physiology: Short-Chain Fatty Acids as Key Bacterial Metabolites. Cell.

[47] Hao Y.T., Wu S.G., Jakovlić I., Zou H., Li W.X., Wang G.T. (2017). Impacts of diet on hindgut microbiota and short-chain fatty acids in grass carp (*Ctenopharyngodon idellus*). Aquac. Res..

[48] Zhou J., Yao N., Wang S., An D., Cao K., Wei J., Li N., Zhao D., Wang L., Chen X. (2019). Fructus Gardeniae-induced gastrointestinal injury was associated with the inflammatory response mediated by the disturbance of vitamin B6, phenylalanine, arachidonic acid, taurine and hy-potaurine metabolism. J. Ethnopharmacol..

[49] Vu S.H., Reyes A.W.B., Huy T.X.N., Min W., Lee H.J., Kim H.-J., Lee J.H., Kim S. (2021). Prostaglandin I2 (PGI2) inhibits *Brucella abortus* internalization in macrophages via PGI2 receptor signaling, and its analogue affects immune response and disease outcome in mice. Dev. Comp. Immunol..

[50] Liu P., Zhu W., Chen C., Yan B., Zhu L., Chen X., Peng C. (2020). The mechanisms of lysophosphatidylcholine in the development of diseases. Life Sci..

[51] Yang S., Chen J., Ma B., Wang J., Chen J. (2022). Role of Autophagy in Lysophosphatidylcholine-Induced Apoptosis of Mouse Ovarian Granulosa Cells. Int. J. Mol. Sci..

[52] Nguyen T.P.L., Nguyen V.T.A., Do T.T.T., Quang T.N., Pham Q.L., Le T.T. (2020). Fatty Acid Composition, Phospholipid Molecules, and Bioactivities of Lipids of the Mud Crab *Scylla paramamosain*. J. Chem..

[53] Overgaard J., Tomčala A., Sørensen J.G., Holmstrup M., Krogh P.H., Šimek P., Koštál V. (2008). Effects of acclimation temperature on thermal tolerance and membrane phospholipid composition in the fruit fly Drosophila melanogaster. J. Insect Physiol..

[54] Nemova N.N., Kaivarainen E.I., Fokina N.N. (2017). Activity of Na+/K+-ATPase and the content of phospholipids in the blue mussel *Mytilus edulis* L. during environmental temperature changes. Appl. Biochem. Microbiol..

[55] Bolstridge J., Fried B., Sherma J. (2010). Effects of temperature on the neutral lipid content ofbiomphalaria glabrataas determined by high performance thin layer chromatography-densitometry and observations on snail fecundity. J. Liq. Chromatogr. Relat. Technol..

[56] Hunsberger A., DeGrandchamp D., Fried B., Sherma J. (2014). Effects of high and low temperatures on the lipid content of the digestive gland-gonad complex of *Biomphalaria glabrata* as determined by high performance thin layer chromatography. J. Liq. Chromatogr. Relat. Technol..

[57] Pérez T., Alba C., Aparicio M., de Andrés J., Quiteria J.A.R.S., Rodríguez J.M., Gibello A. (2019). Abundant bacteria in the proximal and distal intestine of healthy Siberian sturgeons (*Acipenser baerii*). Aquaculture.

[58] Vance J.E. (2008). Thematic Review Series: Glycerolipids. Phosphatidylserine and phosphatidylethanolamine in mammalian cells: Two metabolically related aminophospholipids. J. Lipid Res..

[59] Vance J.E., Tasseva G. (2013). Formation and function of phosphatidylserine and phosphatidylethanolamine in mammalian cells. Biochim. Biophys. Acta Mol. Cell Biol. Lipids.

[60] Li S., Liu Y., Li B., Ding L., Wei X., Wang P., Chen Z., Han S., Huang T., Wang B. (2022). Physiological responses to heat stress in the liver of rainbow trout (*Oncorhynchus mykiss*) revealed by UPLC-QTOF-MS metabolomics and biochemical assays. Ecotoxicol. Environ. Saf..

[61] Lee Y.-S., Kim T.-Y., Kim Y., Lee S.-H., Kim S., Kang S.W., Yang J.-Y., Baek I.-J., Sung Y.H., Park Y.-Y. (2018). Microbiota-Derived Lactate Accelerates Intestinal Stem-Cell-Mediated Epithelial Development. Cell Host Microbe.

[62] Du F., Li Y., Tang Y., Su S., Yu J., Yu F., Li J., Li H., Wang M., Xu P. (2018). Response of the gut microbiome of Megalobrama amblycephala to crowding stress. Aquaculture.

[63] Chen L., Jiao T., Liu W., Luo Y., Wang J., Guo X., Tong X., Lin Z., Sun C., Wang K. (2022). Hepatic cytochrome P450 8B1 and cholic acid potentiate intestinal epithelial injury in colitis by suppressing intestinal stem cell renewal. Cell Stem Cell.

[64] Wang Y., Tao H., Huang H., Xiao Y., Wu X., Li M., Shen J., Xiao Z., Zhao Y., Du F. (2021). The dietary supplement *Rhodiola crenulata* extract alleviates dextran sulfate sodium-induced colitis in mice through anti-inflammation, mediating gut barrier integrity and reshaping the gut microbiome. Food Funct..

[65] Wang D., Ma X., Guo S., Wang Y., Li T., Zou D., Song H., Yang W., Ge Y. (2020). Effect of Huangqin Tang on Urine Metabolic Profile in Rats with Ulcerative Colitis Based on UPLC-Q-Exactive Orbitrap MS. Evid. Based Complement. Altern. Med..

[66] Filimoniuk A., Daniluk U., Samczuk P., Wasilewska N., Jakimiec P., Kucharska M., Lebensztejn D.M., Ciborowski M. (2020). Metabolomic profiling in children with inflammatory bowel disease. Adv. Med. Sci..

[67] Zou Y., Yang Y., Fu X., He X., Liu M., Zong T., Li X., Aung L.H., Wang Z., Yu T. (2021). The regulatory roles of aminoacyl-tRNA synthetase in cardiovascular disease. Mol. Ther. Nucleic Acids.

[68] Lin R., Liu W., Piao M., Zhu H. (2017). A review of the relationship between the gut microbiota and amino acid metabolism. Amino Acids.

[69] Dai Z.-L., Wu G., Zhu W.-Y. (2011). Amino acid metabolism in intestinal bacteria: Links between gut ecology and host health. Front. Biosci..

[70] Aleshin S., Strokin M., Sergeeva M., Reiser G. (2013). Peroxisome proliferator-activated receptor (PPAR)β/δ, a possible nexus of PPARα- and PPARγ-dependent molecular pathways in neurodegenerative diseases: Review and novel hypotheses. Neurochem. Int..

[71] Byndloss M.X., Olsan E.E., Rivera-Chávez F., Tiffany C.R., Cevallos S.A., Lokken K.L., Torres T.P., Byndloss A.J., Faber F., Gao Y. (2017). Microbiota-activated PPAR-γ signaling inhibits dysbiotic Enterobacteriaceae expansion. Science.

[72] Furuta G.T., Turner J.R., Taylor C.T., Hershberg R.M., Comerford K., Narravula S., Podolsky D.K., Colgan S.P. (2001). Hypoxia-Inducible Factor 1–Dependent Induction of Intestinal Trefoil Factor Protects Barrier Function during Hypoxia. J. Exp. Med..

[73] Tian H., Zhu Y., Dai M., Li T., Guo Y., Deng M., Sun B. (2022). Additives Altered Bacterial Communities and Metabolic Profiles in Silage Hybrid Pennisetum. Front. Microbiol..

