# Supplementary material for: Comprehensive Analysis of Microbiome, Metabolome, and Transcriptome Revealed the Mechanisms of Intestinal Injury in Rainbow Trout under Heat Stress
Source: Int J Mol Sci. 2023 May 10;24(10):8569. doi: 10.3390/ijms24108569 (PMC10218511; doi:10.3390/ijms24108569)
Supplement: Supplementary file 1 [file ijms-24-08569-s001.zip › ijms-2356605-supplementary.pdf]

Supplementary Material

Table S1. Primers sequence of QRT-PCR

Gene	Prime sequence (5′-3′)	Product size (bp)
Claudin	F: GGCACGTCTGAGAAACAACA<br>R: TAGGAAGTGGCAGCCTGACT	245
Occludin	F: CAGCCCAGTTCCTCCAGTA<br>R: GCTCATCCAGCTCTCTGTCC	341
ZO-1	F: AAGGAAGGTCTGGAGGAAGG<br>R: CAGCTTGCCGTTGTAGAGG	291
IL-1β	F:ACCGAGTTCAAGGACAAGGA<br>R: CATTCATCAGGACCCAGCAC	181
IL-6	F:CAATCAACCCTACTCCCCTCT<br>R: CCTCCACTACCTCAGCAACC	91
TNF-α	F:GGGGACAAACTGTGGACTGA<br>R: GAAGTTCTTGCCCTGCTCTG	208
IL-10	F: GGATTCTACACCACTTGAAGAGCCC<br>R: GTCGTTGTTGTTCTGTGTTCTGTTGT	135
TGF-β	F: AGATAAATCGGAGAGTTGCTGTG<br>R: CCTGCTCCACCTTGTGTTGT	113
HSP90	F: AGGGTCAAGGAGGTGGTCAA<br>R: AACGAAGAGGGTGATGGGATATC	84
HSP70	F: ATACAAAGCTGAGGATGACGCACA<br>R: ATCTTGCCTTTCATGTTGTCGTCT	114
Cyp8b1	F: AGTACCGCATCAGAGARGGAGACAG<br>R: CGGAAAATTGACATTTACATTCAAAGTG	121
SCD1	F: CCATGGCCTTCCARAAYGAYAT<br>R: CTTGCCCCCACATRTGNGC	124
β-actin	F: TGGGGCAGTATGGCTTGTATG<br>R: CTCTGGCACCCTAATCACCTCT	165

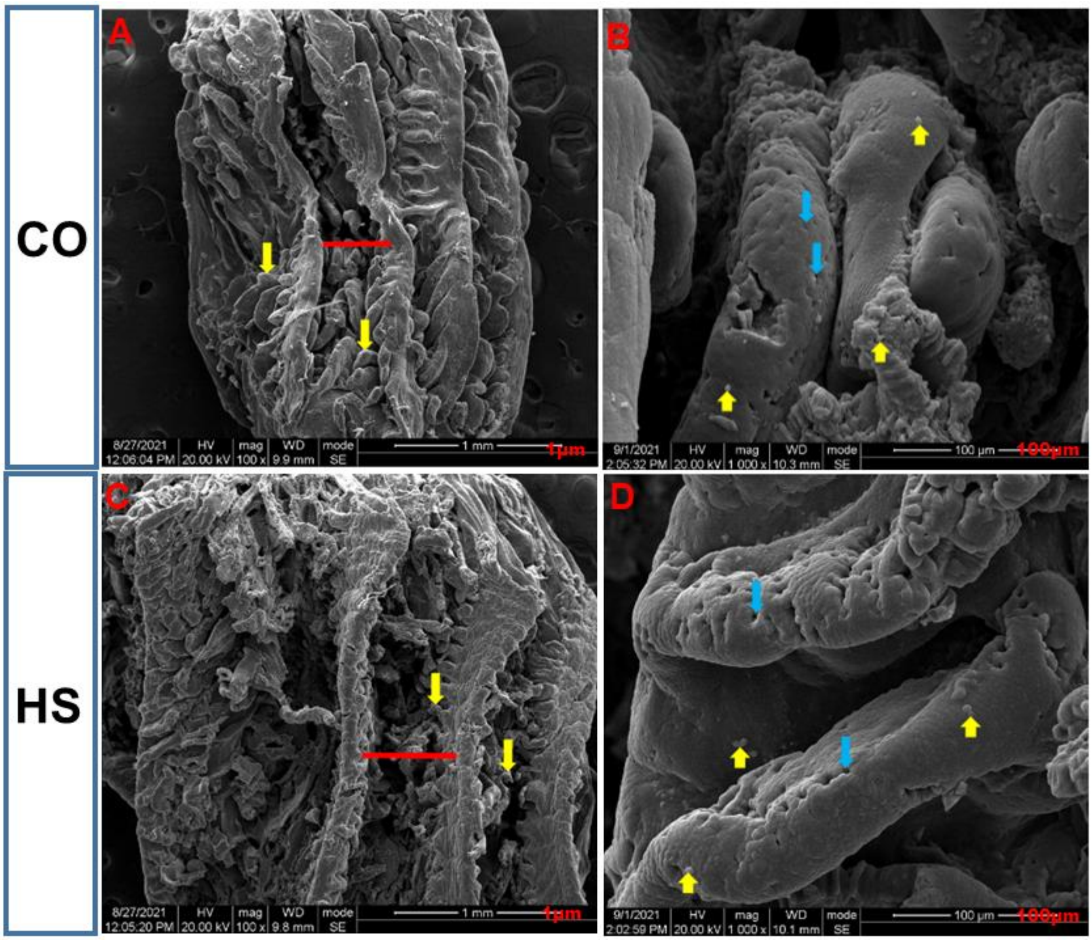

Figure S1. Ultrastructure of distal gut of rainbow trout under heat stress. A-D, the distal gut morphology of the CO group (A-B) and HS group (C-D) was observed under the SEM. Fold distance (—); Bacteria (↑); Secretory pore (↑).
